# Genomic Characterization of Large Heterochromatic Gaps in the Human Genome Assembly

**DOI:** 10.1371/journal.pcbi.1003628

**Published:** 2014-05-15

**Authors:** Nicolas Altemose, Karen H. Miga, Mauro Maggioni, Huntington F. Willard

**Affiliations:** 1 Genome Biology Group, Duke Institute for Genome Sciences & Policy, Duke University, Durham, North Carolina, United States of America; 2 Department of Mathematics, Duke University, Durham, North Carolina, United States of America; The Centre for Research and Technology, Hellas, Greece

## Abstract

The largest gaps in the human genome assembly correspond to multi-megabase heterochromatic regions composed primarily of two related families of tandem repeats, Human Satellites 2 and 3 (HSat2,3). The abundance of repetitive DNA in these regions challenges standard mapping and assembly algorithms, and as a result, the sequence composition and potential biological functions of these regions remain largely unexplored. Furthermore, existing genomic tools designed to predict consensus-based descriptions of repeat families cannot be readily applied to complex satellite repeats such as HSat2,3, which lack a consistent repeat unit reference sequence. Here we present an alignment-free method to characterize complex satellites using whole-genome shotgun read datasets. Utilizing this approach, we classify HSat2,3 sequences into fourteen subfamilies and predict their chromosomal distributions, resulting in a comprehensive satellite reference database to further enable genomic studies of heterochromatic regions. We also identify 1.3 Mb of non-repetitive sequence interspersed with HSat2,3 across 17 unmapped assembly scaffolds, including eight annotated gene predictions. Finally, we apply our satellite reference database to high-throughput sequence data from 396 males to estimate array size variation of the predominant HSat3 array on the Y chromosome, confirming that satellite array sizes can vary between individuals over an order of magnitude (7 to 98 Mb) and further demonstrating that array sizes are distributed differently within distinct Y haplogroups. In summary, we present a novel framework for generating initial reference databases for unassembled genomic regions enriched with complex satellite DNA, and we further demonstrate the utility of these reference databases for studying patterns of sequence variation within human populations.

## Author Summary

At least 5–10% of the human genome remains unassembled, unmapped, and poorly characterized. The reference assembly annotates these missing regions as multi-megabase heterochromatic gaps, found primarily near centromeres and on the short arms of the acrocentric chromosomes. This missing fraction of the genome consists predominantly of long arrays of near-identical tandem repeats called satellite DNA. Due to the repetitive nature of satellite DNA, sequence assembly algorithms cannot uniquely align overlapping sequence reads, and thus satellite-rich domains have been omitted from the reference assembly and from most genome-wide studies of variation and function. Existing methods for analyzing some satellite DNAs cannot be easily extended to a large portion of satellites whose repeat structures are complex and largely uncharacterized, such as Human Satellites 2 and 3 (HSat2,3). Here we characterize HSat2,3 using a novel approach that does not depend on having a well-defined repeat structure. By classifying genome-wide HSat2,3 sequences into subfamilies and localizing them to chromosomes, we have generated an initial HSat2,3 genomic reference, which serves as a critical foundation for future studies of variation and function in these regions. This approach should be generally applicable to other classes of satellite DNA, in both the human genome and other complex genomes.

## Introduction

Long arrays of near-identical tandem repeats, termed satellite DNAs, compose the predominant sequence feature within constitutive heterochromatin in complex genomes [1]. Genomic regions enriched in satellite DNA are often found in or near centromeres [1], [2], and, in some species, they can account for greater than half of the entire genetic material [3]. Yet despite the abundance and potential biological functions of satellite-rich regions, reference assemblies typically exclude them due to the inability of standard assembly algorithms to uniquely align and assemble near-identical tandem repeats.

Efforts to characterize satellite DNA arrays across diverse taxa have proven useful for surveying highly represented copies of tandem repeats, typically reporting a single consensus reference or a small number of representative satellite sequences within a given repeat family [4]–[6]. Only in a few instances have satellite DNAs been studied using a comprehensive inventory of sequences that include all instances of a particular repeat family within the genome, a necessary step for understanding the sequence composition and evolutionary processes that govern sequences within satellite-rich regions of complex genomes [7]. Additionally, attempts to detect and characterize novel tandem repeats are often dependent on long read lengths and on the absence of interspersed stretches of exact sequence identity within each tandem repeat [8]. This presents a considerable problem for characterizing satellite DNA families that are defined by an irregular repeat unit length composed of complex arrangements of short repeats. As a result, this type of complex satellite DNA remains largely uncharacterized, even within the well-characterized human genome. Further, unlike those methods employed to characterize satellite families with well-defined tandem repeat lengths (e.g. [5], [6], [9]), limited sequence-based tools exist to explore the nature of short, irregular repeat sequences across diverse genomic datasets.

Human Satellites 2 and 3 (here, referred to as HSat2,3) define large, heterochromatic blocks adjacent to many centromeric regions in the human genome [10]–[14]. Due to their complex arrangement of divergent 5-bp repeats and the inconsistent lengths of their tandem repeat units, these sequence families remain poorly understood in the human genome, with only a limited sequence characterization of each family collected from a small number of experimentally derived-clones (e.g. [14]–[16]) and from surveys of small stretches of HSat2,3 sequences included in the human reference assembly [17], [18]. HSat2,3 likely originated from an ancestral pentameric simple sequence (CATTC)[14]; however, notwithstanding this commonality, hybridization-based studies [11]–[14], [16], [19], [20] have shown that these two satellite families largely occur in spatially distinct arrays on different chromosomes. Further, characterization of clones derived from different HSat2 and HSat3 sites showed that each array typically has a distinct array length, repeat unit length, and sequence composition [15], [16], [21]–[25]. While some HSat2,3 arrays are nearly identical to arrays on other chromosomes (e.g., on the acrocentric chromosomes [15], [16], [21], [22], [24], [25]), others can be readily distinguished by hybridization-based methods [10], [16], [19]. For example, previous studies determined that the large, multi-megabase array of HSat2 adjacent to the centromere on chromosome 1 is largely defined by a chromosome-specific 1.77 kb repeat unit [16]. Similarly, a male-specific 3.6 kb HSat3 repeat unit comprises the DYZ1 satellite array on the long arm of the Y chromosome [26]–[28]. These limited experimental results suggest the possibility of additional definable subfamilies of HSat2,3 within the human genome, although no study to date has created a comprehensive inventory of these subfamilies using whole-genome sequence datasets.

The length and sequence composition of satellite DNA arrays are known to be variable among individuals, providing a potentially rich source of information to study human variation. The rapid expansion and contraction of satellite arrays by mechanisms such as unequal crossover or sequence conversion [29], [30] is expected to alter the frequency and occurrence of sequence variants between individuals. Consistent with this, previous cytogenetic studies have demonstrated that the large, HSat2,3-rich heterochromatic bands on chromosomes 1, 9, 16, and Y can vary significantly in size among different individuals [31]–[34]. Further, because the DYZ1 array can occupy more than half the length of the Y chromosome [26], variations in the size of this array can dramatically alter the total size of the Y chromosome, and limited studies have suggested that Y-chromosome size may vary across human populations [34], [35].

Although HSat2,3-rich regions of the genome are defined primarily by long, uninterrupted arrays of satellite sequences, several small-scale studies have provided evidence for ‘islands’ of non-satellite sequences that, due largely to their association with satellite-rich regions, are not included in chromosomal reference assemblies [36], [37]. Therefore genome-wide studies of satellite-rich domains may uncover both repetitive and non-repetitive DNA currently missing from reference assemblies.

The absence of satellite-rich regions from reference assemblies prevents detailed study of their potential function(s). In at least some organisms, it is becoming increasingly clear that sequences within these regions can contribute to important cellular functions, such as meiotic chromosome pairing and maintenance of centromere function [38], [39]. Moreover, epigenetic and transcriptional derepression of HSat2,3 sequences is a common feature of cancer cells and stressed cells, coupling the chromatin state of these sequences with genome stability [40]–[45]. Satellite DNAs are also overrepresented in extrachromosomal circular DNAs [46] and in cytoplasmic membrane-associated DNA, a poorly understood class of DNA fragments that have been shown to exit the nucleus and associate with the plasma membrane, where they can be transcribed [47]. Without any sort of reference sequence for satellite-rich regions, however, it is difficult to study their expression and potential regulation at finer scales. Additionally, it is unclear if variation between individuals – in both the satellite and non-satellite components of these regions – has any influence on the function and epigenetic behavior of these regions. A more complete genomic census of satellite DNAs would also aid studies of repeat-associated chromosomal rearrangements, as HSat2,3 sequences are commonly enriched at the breakpoints of Robertsonian translocations [48]–[51].

To address these current limitations, we present here an algorithm to predict HSat2,3 satellite subfamilies and their chromosomal localizations within the human genome, principled on sequence features typical of satellite DNA arrays. For each satellite subfamily, we generate a database of whole-genome shotgun (WGS) reads capturing the full spread of satellite and non-satellite sequences associated with that subfamily, useful as a nonlinear reference sequence for further studies of these regions. We then apply this reference database to short-read WGS sequence data from several hundred individuals to examine HSat3 array size polymorphisms within the human population. This work presents an initial genomic definition of classical HSat2,3 sequences, which underlie the largest heterochromatin-assigned gaps in the human reference assembly, and it offers a framework to study similar satellite-rich regions characteristic of most other complex genomes.

## Results

### Classification of HSat2,3 subfamilies

**Figure 1:**
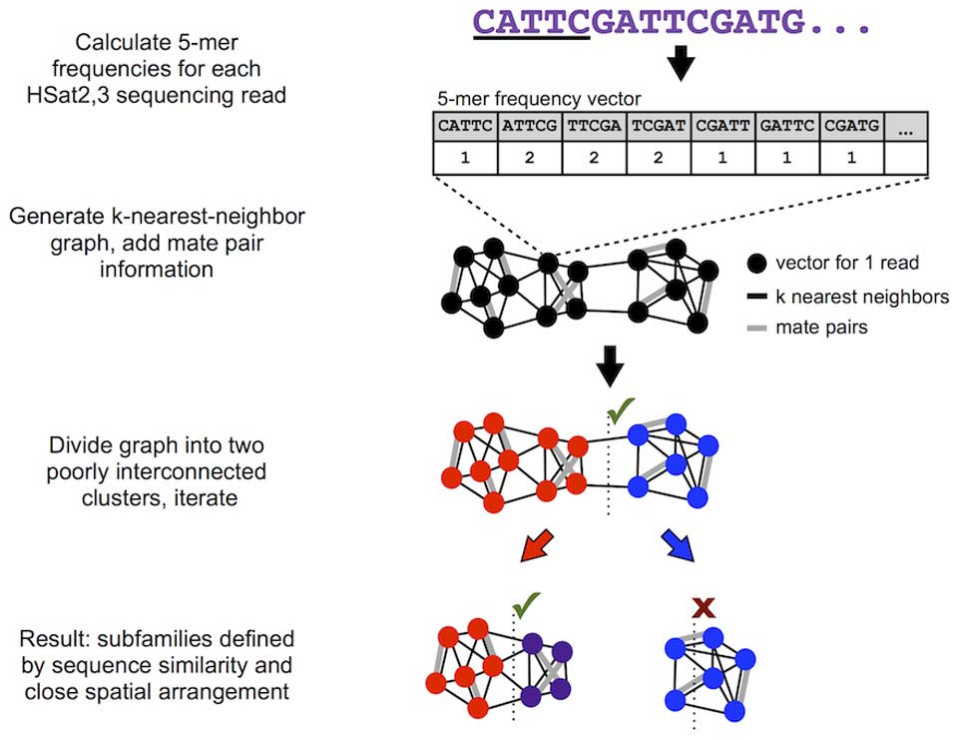
Overview of approach used to characterize satellite sequences. This shows a simplified graphic representation of our overall approach for identifying satellite subfamilies given whole-genome shotgun read data. The actual spectral clustering algorithm is applied in the full 1024-dimension feature space using 50-nearest-neighbor edges weighted according to Euclidean distance.

To build a reference library of all the HSat2,3 sequences present in the human genome, we first applied RepeatMasker [52] to all WGS reads from the genome of a single male donor sequenced at ∼7.8x coverage with paired Sanger sequencing reads [53], yielding a comprehensive database of 396,228 HSat2,3-containing reads (Dataset S1). We verified the comprehensiveness of this database using an independent method that tests for enrichment of the defining CATTC 5-mer on each read (see Methods). Assuming uniform genomic coverage, this database corresponds to roughly 1.5% (48 Mb) of the human genome. To identify shared sequence homology among the HSat2,3-containing reads without direct alignment, we first represented each read as a *k*-mer frequency vector as follows: 1) slide a *k* bp window along each strand-oriented read one base at a time, 2) count the number of occurrences of each distinct *k*-mer, and 3) divide by the length of the read. We chose a *k* of 5 to reduce memory complexity and to reflect the pentameric nature of the ancestral HSat2,3 repeat sequence (CATTC). Additionally, we demonstrate that a modest increase (where *k* = 4–7) provided limited, if any, improvement in our final subfamily classifications (Figure S1). As there are 1,024 (4^5^) possible 5-mers in DNA, we effectively mapped each read onto a vector in a feature space with 1,024 dimensions (Figure 1). In this feature space, reads with similar 5-mer frequencies will tend to be closer together, allowing us to estimate pairwise sequence distance simply as the Euclidean distance between two read vectors (as in [7]). Furthermore, reads mapped onto this feature space can be easily projected in lower dimensions using techniques such as principal component analysis (PCA), allowing one to visualize any predominant clusters.

Next, we performed pairwise comparisons of *k*-mer frequency vectors to identify HSat2,3 reads that are near-identical in sequence composition and thus likely to originate from the same satellite subfamily. Further, as satellite arrays consist primarily of uninterrupted tandem repeats that span regions commonly multiple megabases in size (i.e. greater than the insert length between paired reads), one would expect reads originating from a given satellite array to have mate pairs that predominantly contain similar repeat sequences from the same array. That is, read clusters that define each HSat2,3 satellite subfamily are expected to have high sequence similarity as well as high ‘self-mate-pair’ frequencies. To generate unsupervised read clusters using these criteria, we developed a modified *k*-nearest-neighbor spectral clustering algorithm (see Methods). To identify clusters based on read similarity, we determined the 50 nearest neighbors for each read vector in the 5-mer frequency feature space, resulting in a weighted undirected graph with vertices corresponding to read vectors, edges corresponding to nearest-neighbor relationships in either direction, and edge weights inversely proportional to sequence similarity quantified as the respective normalized Euclidean distance. Importantly, we then overlaid maximally weighted edges corresponding to mate-pair relationships between reads, yielding a graph representing both sequence and mate-pair relationships within the read dataset.

Next, we performed normalized spectral clustering to cut this graph into two large groups while severing the least total edge weight possible (Figure 1) [54]. After this binary split, we then calculated the self-mate-pair frequency of each resulting cluster, which is the proportion of all mate pair edges from each cluster that have both read vectors in the same cluster. We accepted binary divisions resulting in self-mate-pair frequencies >0.8 (shown by paired read simulation to identify satellite arrays greater than 100 kb, as described in Text S1). The resulting daughter clusters were evaluated in the same manner, extending recursive binary divisions until a daughter cluster failed to satisfy the self-mate-pair frequency threshold.

**Figure 2:**
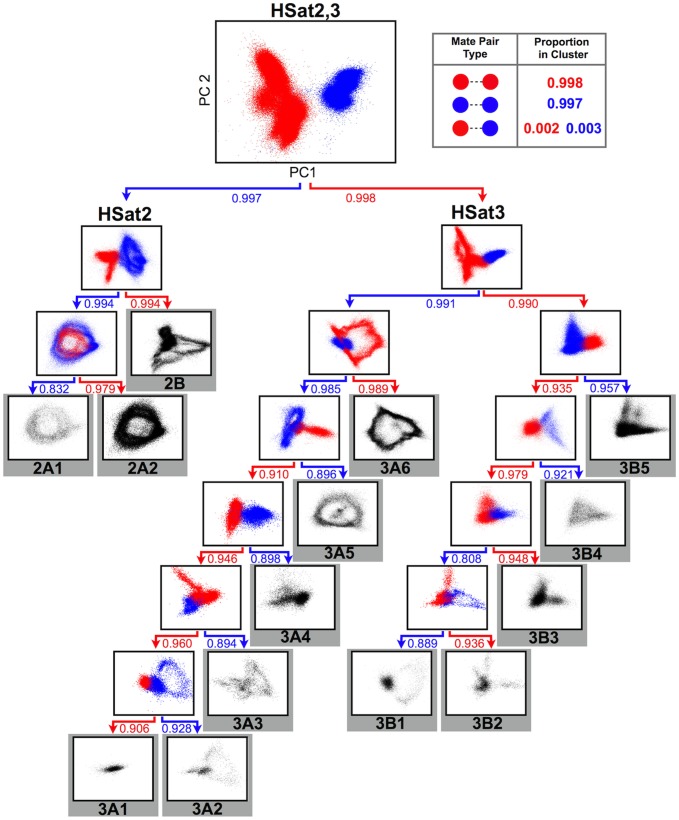
Recursive identification of highly connected subgraphs identifies fourteen HSat2,3 subfamilies. This tree illustrates the iterative binary divisions of the complete HSat2,3 dataset into subfamilies. Each plot is a PCA projection (on principal components 1 and 2) of the normalized 5-mer frequency vectors for all reads in that subgraph. Each point corresponds to one read, colored red or blue by its cluster assignment. Final cluster projections are colored black. The box at the upper right illustrates the concept of self-mate-pair frequencies within the first subgraph division. Arrows below each subgraph are labeled with the self-mate-pair frequency of each daughter cluster, and they are colored to match their cluster of origin in the parent subgraph.

After the initial binary division, we subdivided the read database into HSat2 and HSat3 satellite clusters (as shown in the uppermost panel of Figure 2), with self-mate-pair frequencies of 0.997 and 0.998 (HSat2 and HSat3, respectively), consistent with previous observations that these sequences, although related by an ancestral pentameric repeat sequence, can be distinguished by sequence and spatial organization in the human genome [13]. After recursive binary divisions, this method yielded 14 distinct HSat2,3 subfamilies (three corresponding to HSat2 and 11 to HSat3), and we computed coverage-based estimates of the abundance of each subfamily within the genome (Figure 2, Table 1). Comparisons with previously experimentally verified HSat2,3 clone sequences [15], [16], [21]–[25], [55]–[57] showed high concordance with our predicted HSat2,3 subfamily datasets, indicating that the reported subfamilies comprise genomically distinct subsets, not simply different parts of the same satellite arrays (Table S1). To further evaluate the read clusters corresponding to these subfamilies, we computed the self-mate-pair frequencies of all pairwise combinations of the final fourteen clusters (Table S2). Similar to self-mate-pair frequencies in the initial HSat2 and HSat3 clusters, the majority (10/14) of the subfamily predictions are defined by self-mate-pair frequencies >90% when compared with all other clusters, providing strong support for these cluster definitions. The remaining four subfamilies (HSat2A1, 3A2, 3B1,and 3B2) show somewhat lower self-mate-pair frequencies (73%–83%), likely owing to the fact that these subfamilies share subregions with high homology to other subfamilies (Figure S2 and Table S3).

**Table 1:** Sequence database description of HSat2,3 subfamilies.

Subfamily name	Estimated total size in diploid HuRef genome (kb)	Chromosomal localizations*	Most common subfamily-specific 24-mer
HSat2A1	1,988	1†,10	TTGATTCCATTAGTTTCCATTGGA
HSat2A2	15,366	1†	CATTCGATTCCATTCGATGATAAT
HSat2B	16,233	16†,1,2,7,22	TTCGATTCCATTTGATGATTCCAT
HSat3A1	1,299	10,13,21,22	AATTCCATTCCATTCCATTCGAGT
HSat3A2	1,951	10,13,14,21,22,Y	TACATTCCATTCGACTCCATTCCA
HSat3A3	836	Y†	CTTTACACTCCATTCCTTTCTATT
HSat3A4	2,518	13,14,15,21,22,Y	AATTCCATTCCTTCCCATTCCATT
HSat3A5	4,525	15†	TATTCTATATGATTCCATTCCATT
HSat3A6	12,851	Y†,7	TCGTTTCGATTCCTTTCCATTCCA
HSat3B1	539	not determined	TTGCATTCCACTCGGGTTGATTCT
HSat3B2	961	1†,13,14,21	TTGTTTCAATTCCATTCTATTCCA
HSat3B3	6,109	20†,1,4,5,7,13,14,21,22	TTCCTTTCCAGTTGATTCCATTCC
HSat3B4	1,930	13,14,21,22	TTCCATTCCACTCCACCCGGATTT
HSat3B5	14,279	9†,13,14,20,22	CTCCATTACATTCCATTCCATTCG

† these represent the predominant chromosomal localizations.

*localizations based on flow-sorted chromosome sequence coverage as well as published mappings of sequenced clones and HSat2,3 annotated on hg19 (representing arrays >50 kb).

The resulting HSat2,3 read clusters each represent a deep genomic inventory of the sequences that comprise a physically distinct HSat2,3 subfamily, without use of a consensus sequence. These satellite reference databases are expected to contain essentially all satellite sequence variants as well as representative samples of any non-satellite sequences associated with each subfamily.

### Satellite sequence variation within and between HSat2,3 subfamilies

**Figure 3:**
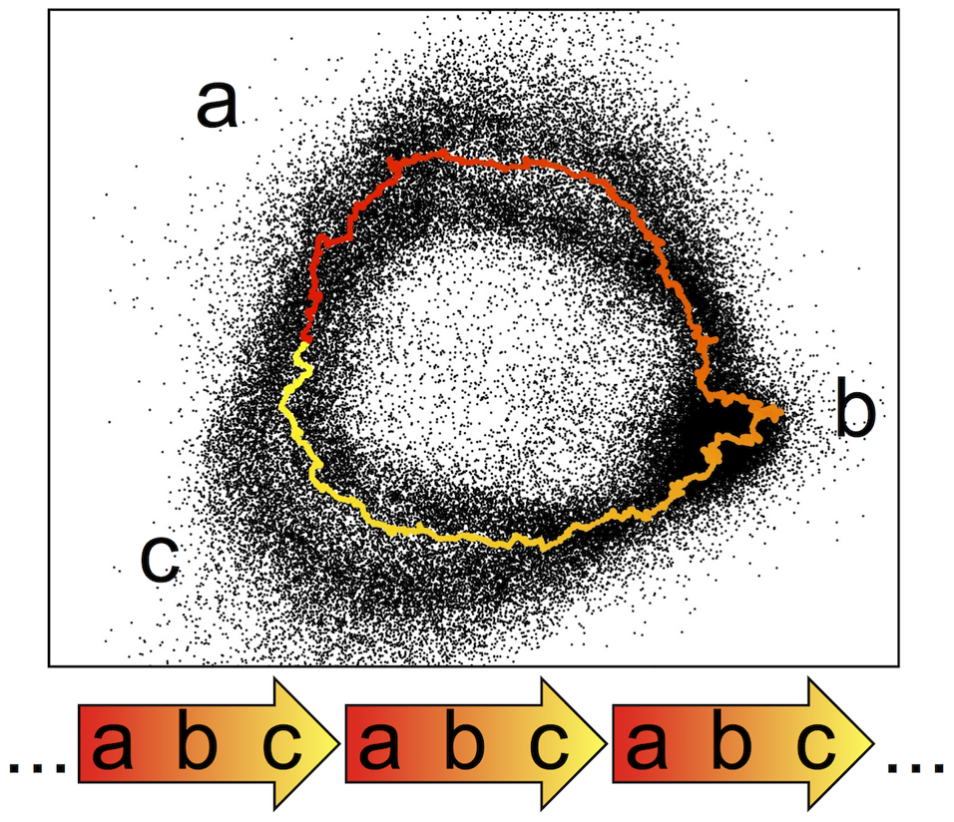
Ring-like topology of HSat2,3 subfamily projections reflects tandem repeat unit organization. Feature vectors representing simulated reads from a 1.77 kb clone sequence from the HSat2 array on chromosome 1 are colored by their starting position on the clone sequence and overlaid on a PCA projection of HSat2A2 reads (black). Arrows below this plot illustrate the tandem nature of the 1.77 kb repeat, which yields the observed ring-like projection as reads are sampled from different subregions of the tandem repeat unit.

To study the sequence features specific to each HSat2,3 subfamily, we analyzed the nature of individual cluster topologies and performed direct sequence comparisons within and between each corresponding satellite reference dataset. Five subfamilies (HSat2A1, 2A2, 2B, 3A5, and 3A6) are observed to have ‘ring-like’ topologies visible in their PCA projections. We hypothesized that reads within these ring-like projections may collectively represent a predominant repeat unit whose length is longer than a single WGS read and whose subregions are sufficiently diverged to be distinguished in the feature space. As depicted in Figure 3, if a highly represented tandem repeat unit, shown subdivided into parts a, b, c, is prevalent, then groups of reads are expected to overlap a-b (red to orange), b-c (orange to yellow), and then c-a (yellow to red), resulting in a circular transition from the end of one repeat unit to the start of the next, found immediately upstream, conceptually similar to the notion of a multimeric, higher-order repeat, defined previously for other satellite DNAs [58]. To test this hypothesis, we simulated a read database from a concatenated array of a published 1.77 kb HSat2 repeat unit sequence corresponding to HSat2A2 [15], [59]. By plotting our simulated data relative to our full sequence database for subfamily HSat2A2 (Figure 3), we show that the general topology corresponds to the presence and prevalence of a tandemly repeating unit. In a similar manner, we demonstrate that the previously published repeat unit for DYZ1 [28] was useful in describing the topology of HSat3A6 (Figure S3a). Notably, many subfamily cluster predictions do not have a similar ring-like topology, perhaps due to short or inconsistent repeat units, as demonstrated for a shorter repeat unit in the HSat3A4 subfamily (1366 bp, pTRS-47, Figure S3b).

Sequence relatedness among individual subfamilies is observed within the hierarchical tree describing their subsequent subgraph divisions (as shown in Figure 2). For example, HSat2A1 and HSat2A2 share a parent subgraph and are closely related on the sequence level, suggesting recent evolutionary sequence divergence. Notably, we show that HSat3 can be divided into two large and very well-separated families (designated as HSat3A,B), each of which is composed of multiple subfamilies. To further estimate the level of similarity between subfamilies, we computed the fraction of overlapping 24-mers between each pair of subfamily read databases. In line with previous observations, HSat2 and HSat3 subfamilies are generally highly distinguishable at this sequence level (i.e. <1% of overlap), with the most prevalent overlap (up to 57%) observed between closely related subfamilies (Table S3). Inversely, this multi-way comparison across all HSat2,3 clusters is useful in predicting those 24-mers that are highly enriched in each subfamily relative to all others (Table 1; Table S4; Dataset S2), making them useful for classifying short sequencing reads among subfamilies and for designing subfamily-specific oligonucleotides for experimental applications.

### Localizing HSat2,3 subfamilies to chromosomes

To estimate the distribution of each HSat2,3 subfamily across the genome, we utilized available HSat2,3 sequences present in the current chromosome reference assemblies (GRCh37) and in Genbank, as well as whole chromosome shotgun (WCS) sequence libraries (representing data from 19 flow-sorted chromosomes [60], [61], Tables S5 and S6).

**Figure 4:**
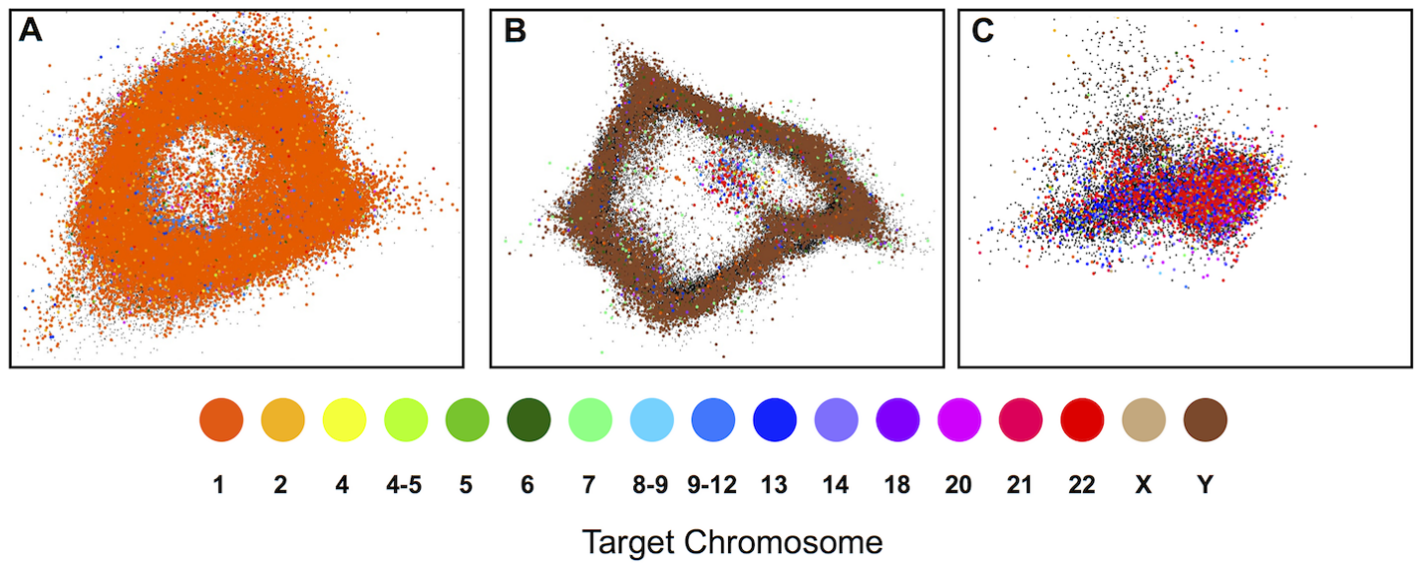
Reads from flow-sorted chromosomes are useful in assigning genome-wide distributions of HSat2,3 subfamilies. Read feature vectors from flow-sorted chromosome datasets (colored according to targeted chromosome(s)) are overlaid on PCA projections of the read databases (colored black) for (A) HSat2A2, (B) HSat3A6, and (C) HSat3A4. The plots qualitatively show enrichment for chromosomes 1, Y, and the acrocentrics, respectively. This enrichment is quantitatively and precisely measured in order to infer the chromosomal localization of each subfamily (see Methods).

To compute the coverage of each HSat2,3 subfamily in each chromosome's WCS dataset, we first computed 5-mer frequency vectors for 186,961 HSat2,3-containing WCS reads, as described earlier, and assigned each one to the same subfamily as its nearest read within the initial reference database. We accounted for experimental contamination from non-target chromosomes using a Bayesian model with an uninformative prior (Table S7 as discussed in Methods). Consistent with the chromosomal assignments of published HSat2,3-containing clones, we observe chromosome 1 enrichment for HSat2A2 [15], chromosome Y enrichment for HSat3A6 [32], and acrocentric chromosome enrichment for HSat3A4 [55], [57] (Figure 4; Table 1). Further, we identified HSat3B5 as the predominant sequence feature of the large, pericentromeric heterochromatic band on chromosome 9, which was previously known to contain HSat3 but lacked any complete clone sequence [15]. We also describe a previously uncharacterized subfamily specific to the Y chromosome, HSat3A3. In line with published high-stringency, hybridization-based mapping experiments, we find that many of our predicted HSat2,3 subfamilies are distributed across more than one chromosome [13], suggesting the possibility of inter-chromosomal sequence homogenization.

In addition to WCS assignments, we studied those HSat2,3 sequences currently present in the human reference assembly (GRCh37/hg19) [62], including those found directly adjacent to heterochromatin gaps (983 kb total), as well as a much smaller number interspersed within chromosome arm assemblies (31 kb total). We assigned these assembly sequences to subfamilies by searching for perfect matches to our subfamily-specific HSat2,3 24-mer libraries (Table S8). Notably, we found that individual subfamilies are observed in small amounts in several locations in the human genome assembly, with the majority observed directly adjacent to (within 500 kb of) the centromeric or heterochromatin-assigned gap [18], [63]. While most HSat2,3 annotations on the assembly are concordant with our WCS-based chromosomal localization predictions, our WCS coverage data lack power to rule out the existence of small (<50 kb) interspersed arrays of HSat2,3 on some chromosomes.

### Localizing unmapped scaffolds to chromosomes

Some genomic regions enriched with HSat2,3 have been shown previously to contain islands of non-satellite sequences missing from the reference assembly [36], [37]. To detect and characterize the presence of such sequences more systematically, we first obtained all unmapped scaffolds from the HuRef assembly that contain at least one uniquely mapping 24-mer (18,780 scaffolds, totaling 43 Mb, including 22 Mb of non-RepeatMasked sequence; see Methods). To determine the chromosomal origin of each scaffold, we used a Bayesian model taking as input sequence coverage from flow-sorted chromosome (WCS) datasets and an uninformative prior (see Methods, Dataset S3). Using this WCS coverage model, we were able to predict the localization of 9,950 HuRef scaffolds to at least one chromosome, totaling 13.7 Mb of non-repetitive sequence. We also localized 555 unmapped scaffolds found in the “decoy sequences” utilized by the 1000 Genomes Project Consortium [64]–[66]. We compared our localizations with available admixture mapping data from two recent studies and observed our chromosomal assignments to be highly concordant (88.89%; 313/352) (Dataset S3). Additionally, we evaluated unmapped scaffolds with reported chromosomal localizations in GenBank (including GRCh37 and GRCh38) and found roughly ∼80% (247/310) of our chromosomal predictions to be concordant. Furthermore, 33/35 unmapped scaffolds with available fluorescence in situ hybridization (FISH) mapping data had concordant chromosomal assignments [53], [67] (Table S10). We observed that the majority of discordant scaffold placements could be attributed to acrocentric chromosome localization (where chromosomes 13,14, 21, and 22 are known to share considerable sequence identity) and/or to low-confidence chromosome localization (e.g. scaffolds that had lower-confidence admixture mapping assignments or lacked whole-chromosome shotgun sequencing data).

**Figure 5:**
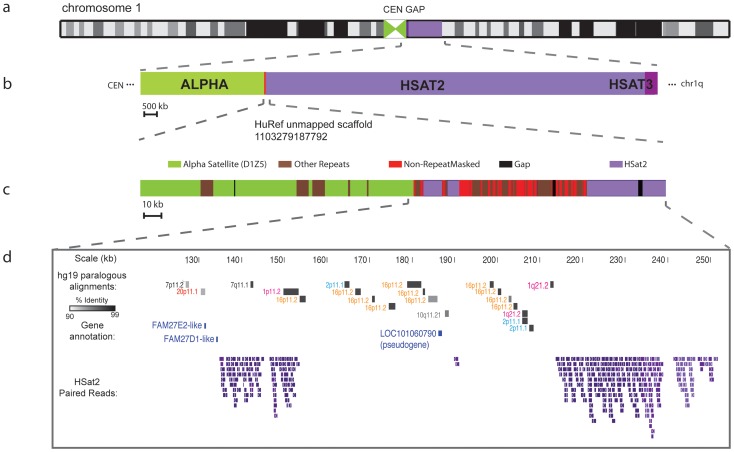
Unmapped scaffold uniquely mapped to HSat2-rich region on chromosome 1. This unmapped scaffold (HuRef SCAF_1103279187792) defines an inter-satellite region in the large centromeric/heterochromatin gap on chromosome 1 (**a**), which is located between alpha satellite (centromeric region) and Human Satellites 2,3 (heterochromatic gap) (**b**). It contains roughly 60kb of non-RepeatMasked sequence (**c**), most of which represents ancient segmental duplications to the pericentromeric regions of chromosomes 1, 2, 7, 10, 16, and 20. Also shown are the positions of annotated gene predictions and HSat2-containing reads used in the assembly of this scaffold (**d**).

We identified 17 large scaffolds (totaling 1.3 Mb of non-repetitive sequence) with three or more mate-pair connections to the same HSat2,3 subfamily, providing evidence for physical proximity to regions enriched in HSat2,3. We evaluated the chromosomal assignments of eleven scaffolds with available mapping results from admixture studies [66], [68] and found them all to be concordant in their chromosomal localizations. Additionally, we report four out of the remaining six scaffolds to have chromosome assignments concordant with available FISH mapping data [53](Table S9). As an example, a single HuRef unmapped scaffold containing roughly 60 kb of segmentally duplicated sequence paralogous to multiple chromosomes was shown to define an inter-satellite sequence region on chromosome 1 by WCS enrichment, PCR-based mapping (Figure S4), and paired-read mapping to HSat2 and adjacent alpha satellite (D1Z5) (Figure 5). Similar to previous studies [36], [37], [53], we observe that the majority (86%) of non-satellite sequences that are associated with HSat2,3 arrays represent low-copy, segmentally duplicated sequences, including eight annotated gene predictions (Table S9). The introduction of inter-satellite regions is expected to improve the completeness of ongoing genomic studies aimed to understand the evolution and biological functions of these sequence paralogs.

### Estimating array size using low-coverage, short-read sequencing data

Satellite arrays have been shown previously to vary in size within the human population [31]–[34], [69]–[71]. Therefore, to illustrate the utility of our HSat2,3 reference database for studying satellite array size variation using short-read sequencing data, we investigated the total size of the Y-chromosome heterochromatic locus classically known as DYZ1 (corresponding to subfamily HSat3A6) using genomic sequence data from 396 male individuals from the 1000 Genomes Project [64], [65]. Predicted DYZ1 array sizes (see Methods, Table 2, Dataset S4) varied over an order of magnitude (7-98 Mb, with mean 24 Mb), consistent with previous experimental observations of Y-chromosome length variability [27], [70], [71].

**Table 2:** Array size estimates for Y-haplogroups.

Y haplogroup	No. samples	HSat3A6 array size estimate in Mb, mean +/− s.d. (range)
B	2	18.9+/−4.1 (16.0–21.9)
C	5	27.7+/−8.8 (14.3–36.0)
D	11	20.6+/−7.6 (14.5–41.4)
E	94	22.7+/−10.1 (10.0–61.3)
G	5	21.6+/−8.1 (14.7–34.5)
I	25	27.6+/−14.4 (14.3–83.0)
J	15	25.9+/−7.6 (13.1–44.1)
N	20	35.0+/−21.7 (17.8–98.4)
O	89	29.3+/−10.8 (10.8–58.5)
Q/P	9	14.4+/−2.2 (11.5–18.1)
R	114	20.3+/−9.0 (7.1–67.4)
T	7	12.8+/−4.3 (8.5–18.8)

**Figure 6:**
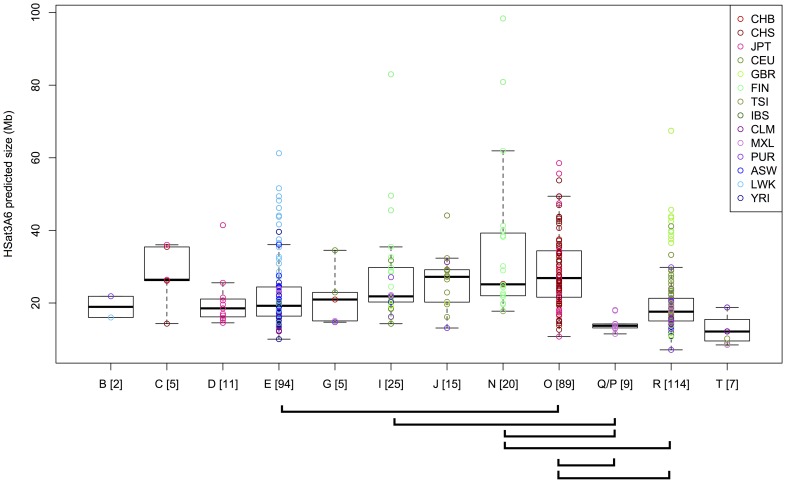
HSat3A6 (DYZ1) array size estimates in 396 individuals. Each circle represents an HSat3A6 size estimate for a single individual, and it is colored by the population designation of that individual. Individuals are grouped by Y haplogroup assignments, and boxplots illustrate the distribution of array sizes within each haplogroup. Brackets below the plot indicate pairs of haplogroups with p<0.001 in a pairwise, two-sided, two-sample Wilcoxon rank-sum test (with Holm correction for multiple testing), indicating a location shift in the distributions of array sizes between these haplogroups.

Because HSat3A6 is on the non-recombining portion of the Y chromosome, it is inherited as a single haplotype linked with previously characterized sequence polymorphisms that distinguish Y haplogroups (reviewed in [72]). Thus, one predicts that different haplogroups would contain different HSat3A6 array length distributions given their independent evolutionary histories since splitting. Further, the variability within each haplogroup allows one to observe how rapidly satellite array lengths evolve on the time scales since Y haplogroup divergence. As illustrated in Figure 6, variable HSat3A6 array length distributions are observed both within and between haplogroups, consistent with a model of rapid satellite array length evolution. To test whether array size distributions differ significantly within any haplogroup compared with all other haplogroups, we performed a two-sided, two-sample Wilcoxon rank-sum test for each pairwise combination of haplogroups, using a Holm correction for multiple testing. Six pairwise combinations show highly significant location shifts (p<0.001). For example, array sizes in the Asian-enriched O haplogroup are stochastically greater than array sizes in the African-enriched E haplogroup (p = 3.05×10^-7^), with a median difference of 7.6 Mb. Notably, the largest array sizes observed occurred within Finnish males (in haplogroups I, N), but other samples with large arrays appear in haplogroups enriched in Asian and African populations (E,O,R).

## Discussion

Achieving more complete reference genome assemblies will require better characterization and representation of heterochromatic regions, which are typically composed of long arrays of satellite repeats along with embedded non-satellite sequences. Here, we present a computational framework for studying both the satellite and non-satellite components of heterochromatic genomic regions, optimized for satellite families that are composed of a complex arrangement of simple repeats, a common feature of satellite families across diverse taxa. Within this study, we have focused in particular on Human Satellites 2 and 3, which are composed of diverged arrangements of 5-bp repeats and constitute roughly 1.5% of the human genome, typically occupying heterochromatic blocks adjacent to centromeric regions. We used an alignment-free approach to detect and cluster highly similar HSat2,3 sequences among genome-wide collections of WGS paired reads, yielding 14 subfamilies distinct in sequence composition and physical location in the genome. Further, by utilizing sequence libraries of isolated human chromosomes, we predict the genomic distribution of these subfamilies, presenting chromosome-assigned satellite reference databases capable of describing the sequence variation within multi-megabase HSat2,3 arrays. Our computational framework is broadly applicable for clustering satellite DNA paired-read libraries, which can be initially compiled from WGS data according to their enrichment of simple repeat patterns (in our case, ‘CATTC’). Further application of our strategy to less characterized, or novel satellite DNA families is possible by performing an initial survey of short repeat enrichment in a given genome using methods presented here and/or by employing existing software designed for short repeat discovery [4], [8] to generate an initial read database for subfamily classification.

As an extension of these reference genomic databases, we developed a library of HSat2,3 subfamily-specific 24-mers useful for estimating the size of each subfamily across short-read, whole-genome datasets of individual genomes from diverse human populations. Frequent non-homologous sequence exchange is expected to promote rapid expansion and contraction of repeat variants within each satellite DNA array. Focusing on a single, haploid HSat3-rich region on the long arm of the Y chromosome, we provide satellite array length estimates, identifying a substantial difference in array length across 396 male individuals from distinct Y haplogroups. Moreover, we demonstrate that this variation is observed within closely related members of the same haplogroup, illustrating the potential for recent sequence expansion and contraction within these sites.

This analysis and the methods described herein provide an opportunity to characterize not only satellite DNA but also non-satellite DNA sequences found in close proximity to each HSat2,3 subfamily. In doing so, we have localized roughly 1.3 Mb of previously unmapped HSat2,3-associated sequences to particular chromosomes, providing a new potential source of assayable polymorphic markers to tag the heterochromatic gaps of the human genome for studies of recombination, population genetics, and disease association. These scaffold sequences, along with our satellite reference database and our library of subfamily-specific 24-mers, also provide potential mapping targets to study the epigenetics and expression of these poorly characterized regions of the human genome. This work thus serves as an initial reference database for HSat2,3-enriched regions within a single diploid genome, providing a foundation for ongoing genomic studies aimed at understanding their potential biological function and cellular regulation.

## Methods

### HSat2,3-containing sequencing reads

We downloaded all ∼32M unassembled WGS sequencing reads generated by the HuRef genome project [53] and we processed them using RepeatMasker ([52]; parameters: slow, cross-match). Candidate HSat2,3-containing reads were identified as having RepeatMasker annotations matching “HSATII,” “(CATTC)n,” or “(GAATG)n”. These represent the canonical reference sequences for HSat2 and HSat3 (note: the latter 2 repeat types are simply reverse complements representing HSat3). A total of 455,513 reads had at least one match to these repeat types, with the lowest percent identity match of any annotated segment on any read being 61.4%. The mean number of matched bases per read was 701, compared to the average HuRef read length of 877 base pairs, consistent with the expectation that reads derived from long, contiguous domains of HSat2,3 will tend to contain HSat2,3 along their entire length. Of these reads that have mate pairs (∼65%), 93.3% have mate pairs also containing HSat2,3, consistent with the expectation that mate pairs derived from long contiguous domains of HSat2,3 will tend to both contain HSat2,3.

To evaluate the comprehensiveness of this dataset, we employed an independent method of identifying HSat2,3-containing reads, in which we calculated the enrichment above background of the ancestral HSat2,3 5-mer, CATTC, on each read. First, we counted the number of occurrences of CATTC (or GAATG, whichever was more frequent) on each read. To model the background expectation of the number of CATTC occurrences on each read, we used a Poisson distribution with parameter 2.37 (the observed mean number of CATTC on each read). Using this background model, we selected a threshold expected to yield 1 false positive in the entire HuRef read dataset of ∼32 million reads, classifying any read with at least 14 occurrences of CATTC/GAATG as HSat2,3. We found the resulting dataset to be 97% concordant with the RepeatMasker dataset (excluding only 1.8% of the RepeatMasker dataset and adding only 5244 new reads). Given this high concordance, we utilized the RepeatMasker dataset for downstream analyses.

To examine further whether there are diverged HSat2,3 subfamilies not detected by the canonical RepeatMasker consensus sequences or by CATTC enrichment, the 455,513 candidate HSat2,3-containing reads were scanned for matches to any other tandem repeats present on at least 100 reads. Tandem repeats that represent 1-away variants of the canonical CATTC repeat (e.g. CACTC, CATTT, TGGA, etc.) were then identified among all HuRef reads. This yielded 168,455 new reads, but with a mean of only 76 bp matching a “variant” repeat per read. Moreover, only 2% of these reads had mate pairs to other reads matching any of these variants. Because these properties suggest that these variant tandem repeats do not occupy large satellite arrays, these reads were not included in any further analysis, as they most likely represent spurious matches to sequence unrelated to HSat2,3. As an exception, we did include 531 variant-matching reads that are paired with reads matching the canonical repeats. Next, we eliminated 998 redundant reads (duplicate reads taken from the same clone end) from this set, and further eliminated 16,748 reads with fewer than 20 continuous bases matching HSat2,3. We also set aside 1,738 reads matching both strands of the HSat2,3 consensus sequence, as these may represent inversion breakpoints that would confound the clustering analysis.

We reverse complemented reads to be in the same orientation (the strand predominantly containing CATTC), and we masked out all bases not matching HSat2,3 as well as all low-quality bases (phred quality scores less than 20). To recover any short, diverged HSat2,3 subsequences that may have been excluded from RepeatMasker alignments on these reads, we unmasked any short (<100 bp) stretches of high-quality, non-HSat2,3 bases. Finally, we eliminated those reads with fewer than 75 continuous bp of high-quality, HSat2,3-matching sequence.

This yielded 396,228 nonredundant sequences matching HSat2,3, with an average number of unmasked bases per read of 544 out of an average trimmed read length of 571 bp. These HSat2,3 reads represent 1.5% of all 29,097,487 HuRef reads satisfying the final quality cutoff (*≥*75 bp continuous bases with phred *≥*20), providing a rough estimate of the abundance of HSat2,3 in the genome. Of the HSat2,3-containing reads in this set that have high-quality mate pairs (*≥*75 bp continuous bases with phred *≥*20), we calculated that 95% have mate pairs belonging to this set.

### HSat2,3 subfamily prediction

Each HSat2,3-containing HuRef read was processed into a composition vector by counting the frequency of each possible 5-mer across all HSat2,3-annotated segments within that read. Composition vectors for all reads were stored in a 396228×1024 matrix, for which rows were rescaled to sum to 1, then columns were rescaled to have mean 0 and variance 1. Fifty nearest neighbors were then computed for each composition vector and recorded as a weighted undirected graph, with edge weights corresponding to the Euclidean distance between nearest neighbors. Mate pair relationships were added as additional edges and given maximum weight, yielding a weighted combined graph. Binary spectral clustering was performed iteratively on this graph until the self-mate pair frequency of one or both child clusters fell below an empirically determined threshold of 0.8 (see Text S1).

### Chromosomal localization of unmapped scaffolds

We obtained all unmapped scaffolds from the HuRef assembly [53] and identified uniquely mappable, non-RepeatMasked 24-mers along each scaffold sequence. WCS reads were quality masked at a phred score threshold of 20 and then aligned using BWA-SW [73](with parameter “-z 1”) to the HuRef assembly (including all unmapped scaffolds). We discarded all WCS reads whose alignments had a mapping quality of 0 and all reads that lacked an exact match to one or more unique 24-mers. Using a Poisson likelihood model and a uniform prior, we then calculated the posterior probability that each scaffold originates from each chromosome given its WCS coverage and mappability (see Text S1 for detailed model specification).

### Chromosomal localization of HSat2,3 subfamilies

To account for the fact that each WCS sample is not a pure representation of the target chromosome, we created a Bayesian model to estimate the most likely amount of each HSat2,3 subfamily on each chromosome. The model treats each HSat2,3 subfamily independently. For each subfamily, it takes as input the number of reads in that subfamily for each WCS sample (***y***), a ‘mixing vector’ to estimate the proportion of reads originating from each chromosome in each WCS sample (***X***), and a distribution representing prior beliefs about ***β*** **,** the actual amount of that HSat2,3 subfamily on each chromosome (the parameter we would like to estimate).

We estimate ***y*** by first processing all WCS reads in the same manner as the HuRef reads, identifying all HSat2,3-containing reads and representing them as scaled 5-mer frequency vectors. For each WCS read, we compute its nearest neighbor among all HuRef HSat2,3-containing reads and assign it to the same subfamily. We estimate ***X*** by computing for each WCS sample the read coverage across all valid unique bases on each chromosome (see Text S1 for specification of valid unique bases). We then multiplied these coverage values by the estimated total size of each chromosome in hg19, yielding the total number of reads that would be expected to map to that chromosome if the entire chromosome were uniquely mappable, and we divided by the sum across all 24 chromosomes to yield an estimated proportion of reads from each chromosome. Finally, we used a relatively uninformative prior for ***β***, specified as independent truncated uniform distributions for each chromosome. With this prior, the proportion of each HSat2,3 subfamily on each chromosome has an equal prior probability of being any value in the interval (10^−8^, 0.5). The upper bound indicates that no HSat2,3 cluster may occupy more than half the length of a chromosome. This upper limit serves to reduce wasteful sampling from very unlikely large values. The lower limit is set to avoid computational errors stemming from values equal to or close to 0. This uninformative prior results in posterior estimates that rely almost entirely on the sampling model.

Our sampling model for each HSat2,3 subfamily for each WCS sample *i* is: ***y*** *_i_* | ***β*** ∼ binomial(*n_i_*, ***X_i_*** ** ***β***). That is, for a given HSat2,3 subfamily, the sampling model treats the number of reads from that subfamily in each WCS sample as the result of a binomial draw from a pool of chromosomes mixed according to the sample's mixing vector. Specifically, for a given WCS sample, the probability of drawing a read from a particular HSat2,3 subfamily is equal to the sum of the probabilities of drawing a read belonging to that cluster from each of the 24 chromosomes. This depends both on the mixing vector in **X** and on the estimated proportion of each chromosome belonging to that HSat2,3 cluster (***β*** **)**. The total likelihood is the product of the likelihoods calculated for each WCS sample. Thus, for a particular HSat2,3 cluster, one can calculate the total likelihood of seeing the observed read counts in all 32 WCS samples (**y**) given the fixed mixing vectors in ***X*** and an estimate of the proportion of each chromosome belonging to that cluster (***β*** **)**.

To estimate the most probable posterior values of ***β*** according to this model, we used the WinBUGS software package to sample from the posterior distribution of ***β*** [74]. For each HSat2,3 cluster we generated four Markov Chain Monte Carlo (MCMC) chains each with 16,000 iterations, and we thinned these to 3,000 posterior samples after a 1,000-iteration burn in for each chain. On untargeted or mixed-targeted chromosomes, we discard results discordant with published mapping information from clone sequences and assembly sequences (see Text S1).

### Subfamily-specific *k*-mer library

HuRef reads assigned to each of the 14 subfamilies were scanned for high-quality 24-mers (phred score >20 for all bases). For each subfamily, a set of “subfamily-specific 24-mers” were identified as those present in at least 1% of HuRef reads within that subfamily and in no more than 0.1% of reads in any other subfamily. We also required that these subfamily-specific 24-mers not be found in any HuRef reads lacking annotated HSat2,3.

### Array size estimates

Low-coverage Illumina sequencing reads produced by the 1000 Genomes Project from 400 male individuals and 414 female individuals from 14 diverse populations were obtained [64], [65]. Reads were scanned for high-quality 24-mers (phred score >20 for all bases), which were compared with the 2,696 subfamily-specific 24-mers for HSat3A6. For each individual the total number of high-quality 24-mers across all reads and the total number of high-quality 24-mers exactly matching an HSat3A6-specific 24-mer were enumerated. For each male individual, the HSat3A6 array size was first estimated as follows: (proportion of all HQ 24-mers matching an HSat3A6-specific 24-mer)/(0.459, the proportion of all HQ 24-mers on HSat3A6-containing HuRef reads matching one of the HSat3A6-specific 24-mers)*(5,976,710,698 bp, the estimated size of the diploid male genome from hg19).

To correct for any coverage bias, a set of 49,994 unique 24-mers was obtained from the chrY reference sequence in hg19. These control 24-mers were matched to have the same distribution of AT-richness as the HSat3A6 24-mers. The coverage of these control 24-mers was enumerated in the 814 individuals in the same manner as the HSat3A6-specific 24-mers. A total of 616 control 24-mers, corresponding to the bottom and top 0.5% of coverage across all male individuals or to 24-mers with excessive coverage (>10 total reads) in female samples, were eliminated to increase the likelihood that the remaining set were truly single-copy, Y-chromosome-specific 24-mers.

To estimate the total error in the array size estimates, the size of the control region was estimated by the same calculation for each male individual and compared with the actual value (49,378 bp). All sample size estimates were corrected down by the mean error rate (5% overestimation), probably representing AT-rich coverage bias. After this correction, 95% of male samples had control size estimates within 12% of the actual value. Four outlier samples (control error >30%) were eliminated from downstream analysis.

Chromosome Y haplogroup assignments for each individual (obtained through personal correspondence, C. Tyler-Smith) were used to evaluate whether different haplogroups had significantly different array size distributions compared with all others. Each haplogroup was compared with each other haplogroup using a two-sided, two-sample Wilcoxon rank-sum test with a Holm correction for multiple testing.

## Supporting Information

Figure S1 **Evaluation of increased** ***k*** **-mer length to resolve HSat3B (1,2,3) subfamilies.** Projections of HSat3B read frequency vectors (from the subgraph preceding subdivisions into HSat3B1, 2, and 3 subfamilies, referenced in Figure 2 in our main manuscript) are shown at varying lengths of k (where *k* = 4 (a), k = 5 (b), k = 6 (c), and k = 7 (d)). Reads previously assigned to HSat3B3 (with a 94.8% self-mate-pair frequency provided in our original study) are shown in blue, and the subsequent binary division, consisting of reads from HSat3B1 and 2 (with an 80.8% self-mate-pair frequency), are shown in red.
(TIF)

Figure S2 **Visualization of cluster-bridging reads between 3 highly connected clusters.** Top: 3D PCA projection (principal components 4,5,6) of HSat2A1 (red) and 2A2 (blue). 2A1 reads pairing to 2A2 are colored magenta, and their paired 2A1 reads are colored cyan. Middle: 3D PCA projection (principal components 1,2,3) of HSat3A5 (red) and 3A2 (blue). 3A5 reads pairing to 3A2 are colored magenta, and their paired 3A2 reads are colored cyan. Bottom: 2D PCA projection (principal components 1,2) of HSat3B1 (red) and 3B3 (blue). 3B1 reads pairing to 3B3 are colored magenta, and their paired 3B3 reads are colored cyan. For 3D plots, a perspective was selected to maximize the visible distinction of cluster-bridging reads. This distinction illustrates the fact that cluster-bridging reads may represent subregions of close homology between clusters.
(TIF)

Figure S3 **Additional clone sequences overlaid on PCA projections.** Reads were simulated from complete clone sequences chrY DYZ1 (a) and pTRS-47 (b) and overlaid on PCA projections of HSat3A4 and HSat3A6, respectively (black points), colored by their start positions on each clone sequence.
(TIF)

Figure S4 **Somatic Cell Hybrid Panel Sequence-tag site mapping of unmapped scaffold localized to the centromeric region on chromosome 1.** PCR was performed on a panel of DNA from human-rodent hybrid cells containing a single human chromosome each. We used a set of primers designed to be unique to SCAF_1103279187792 and demonstrate amplification only in the samples containing human chromosome 1. “HLII”: HyperLadderII; “1-24,X,Y”: rodent-human hybrid DNA samples containing each human chromosome in a rodent background; “H1,H2”: positive control whole-genome DNA from human donors; “M1,M2”: negative control whole-genome DNA from mouse background; “C1,C2”: negative control whole-genome DNA from Chinese hamster background; “V”: positive control whole-genome DNA from the HuRef donor individual; “-”: no DNA negative control.
(TIF)

Table S1 **Comparisons of HSat2,3 subfamilies with published clone sequences.** Each clone sequence was scanned for exact matches to subfamily-specific 24-mers from each HSat2,3 subfamily and assigned accordingly.
(PDF)

Table S2 **Proportion of self-paired reads in all pairwise subfamily comparisons**. Each self-mate-pair frequency value is calculated as (A→A)/(A→A +A→B), where A and B represent the two clusters being compared and A→B represents the number of mate pairs from A that belong in cluster B. Self-mate-pair frequencies are shaded according to their values, with lower values shaded red. Above: a tree illustrating the subgraph divisions used to generate the final fourteen clusters, as a point of comparison.
(PDF)

Table S3 **Proportion of redundant 24-mer overlap in all pairwise subfamily comparisons.** Each value represents the proportion of all redundant 24-mers in the smaller subfamily's reference database that are found in the larger subfamily's reference database (without replacing 24-mers in the larger database that have already been counted). Intuitively, this estimates the proportion of the smaller cluster that is ‘contained in’ the larger cluster. Values are colored yellow to red from low to high. Along the diagonal is the fold compression within each subfamily, which is the fold reduction in the number of 24-mers when redundant 24-mers are eliminated, a measure of each cluster's self-similarity.
(PDF)

Table S4 **Subfamily-specific 24-mers identified within each subfamily.** Column 1 lists the number of high-quality (all bases have phred>20) 24-bp windows across all reads in each subfamily's read database (note: this is limited to 24-bp windows matching 24-mers seen on at least 2 reads in the entire HSat2,3 dataset, to reduce 24-mers likely to contain sequencing errors). Column 2 lists the total number of non-redundant 24-mers across all of these windows. Column 3 lists the “fold compression,” calculated as the quotient of Column 1 and Column 2, a measure of the self-similarity of the sequences in each cluster. Column 4 lists the number of 24-mers that are subfamily-specific (defined as present on >1% of reads in that subfamily and <0.1% of reads in any other subfamily, and on no non-HSat2,3 reads). The last column lists the proportion of 24-bp windows (from Column 1) that match a subfamily-specific 24-mer.
(PDF)

Table S5 **WCS datasets used in this analysis.** Each row represents one of the 32 whole chromosome shotgun datasets used in this analysis, listing the target chromosome, the ID of the donor individual, the sex of the donor, the sequencing center (SC = Sanger Center, WUGSC =  Washington University, WIBR  =  Broad Institute), the total number of Sanger sequencing reads, and the search terms required for downloading each dataset from the NCBI trace archive.
(PDF)

Table S6 **Mixing matrix estimating percent of WCS reads from each chromosome for each sample.** Each row represents one WCS dataset and lists the estimated percentage of reads from each chromosome based on coverage of unique 24-mers on each chromosome. The last row gives the relative sizes of each chromosome for comparison. Column labels shaded light grey correspond to chromosomes present only in mixtures with other chromosomes, and those shaded dark grey are the 5 chromosomes untargeted by any of the available datasets. Percentages greater than 75 are shaded yellow; those between 20 and 75 are shaded orange and correspond to mixed chromosome datasets.
(PDF)

Table S7 **HSat2,3 localization predictions from WCS data.** For each subfamily and each chromosome, this table lists the median abundance estimate (in kb) of 3,000 samples from the posterior distribution along with the boundaries of a 95% highest posterior density interval. Confident estimates above 50 kb are colored according to size, yellow being smallest and red being largest. Shaded grey are two potentially artifactual results inconsistent with hybridization-based studies for the localization of these subfamilies. Row labels shaded light grey correspond to chromosomes present only in mixtures with other chromosomes, and those shaded dark grey are the 5 chromosomes untargeted by any of the available datasets. Some of these untargeted chromosomes still obtain confident abundance estimates given information inferred from their varying contamination of WCS datasets targeting other chromosomes.
(PDF)

Table S8 **HSat2,3 annotated on hg19 assembly fragments.** This table lists all clones in the hg19 assembly that contain at least 1 kb of HSat2,3 as annotated by RepeatMasker. The fourth column designates whether each clone is centromere/heterochromatin proximal (within 500 kb) or located on the chromosome arms. The last column indicates the HSat2,3 subfamily assignment of each region based on perfect matches to subfamily-specific 24-mers.
(PDF)

Table S9 **Large HSat2,3-associated unmapped scaffolds localized by WCS coverage.** For each of the seventeen HSat2,3-associated large unmapped scaffolds, this table lists the total non-gap, non-RepeatMasked length (column 3); the number of unique 24-mers used for WCS mapping (column 4); the percentage of non-RepeatMasked bases in continuous stretches >500 bp that align to hg19 at >90% ID and >500 bp (column 5); the names of genes and pseudogenes (labeled (p)) annotated in GenBank (column 6); the chromosomes assigned by WCS coverage mapping in this study (column 7); the chromosomal bands assigned by Genovese *et al.* 2013 where applicable (column 8); the primary and secondary chromosomal bands assigned by fosmid FISH by Levy *et al.* 2007 (column 9); the number of HSat2,3 reads used to assemble the scaffold, by subfamily (column 10); the number of HSat2,3 reads paired with reads used to assemble the scaffold, by subfamily (column 11).
(PDF)

Table S10 **Experimental validation of HSat 2,3 associated unmapped scaffolds with reference to the Cytogenetic Resource of FISH-mapped, Sequence-tagged Clones.** To provide further experimental validation of our unmapped scaffold chromosome assignments (listed in column 1), we compare our localizations with the reported mappings of 35 FISH-mapped clones available from the Cytogenetic Resource of FISH-mapped, Sequence-tagged Clones, or CytoBAC, (The BAC Resource Consortium (2001) Nature 409:953-958) and Levy *et al.* 2007). We highlight concordant predictions for 33/35 of the FISH assignments in red, indicating agreement in localization between our predictions (column 2) and those observed in previous experimental studies (column 3).
(PDF)

Text S1 **Provides supplemental methods and corresponding references: simulating self-mate-pair frequencies for contiguous sequence domains, spectral clustering of feature vectors, and localization of unmapped scaffolds using WCS data.**
(PDF)

Dataset S1 **Read trace identifiers assigned to each HSat subfamily. SD1_HuRefHSat23readInfo.txt:** This file lists the trace identifiers and subfamily assignments for all 396,228 HuRef HSat2,3 reads included in this study. Paired reads are listed in the same row. Column 1 lists the TI number of an HSat2,3 read, and Column 2 lists the subfamily assignment of that read. If the read in Column 1 is paired, Column 3 lists the TI number of its mate pair; otherwise, this column is assigned “NaN”. If the read in Column 3 is also an HSat2,3 read, Column 4 lists its subfamily assignment; otherwise (or if there is no mate pair), this column is assigned “NaN”. Each read is listed only once in this file. This supplemental data file is available in the Dryad Digital Repository: doi:10.5061/dryad.vg885.
(ZIP)

Dataset S2 **Subfamily-specific 24-mers. SD2_SubfamilySpecific24mers.txt:** This file lists all subfamily-specific 24-mers (Column 1) along with their subfamily assignments (Column 2). A subfamily-specific 24-mer is defined as a 24-mer that occurs on >1% of HuRef reads within a given HSat2,3 subfamily and on no more than 0.1% of reads in any other subfamily (and on no non-HSat2,3 reads). Column 3 lists the proportion of reads containing that 24-mer in that subfamily, and Column 4 lists the proportion of High Quality (phred>20 for all bases) 24-bp windows matching that 24-mer in that subfamily. This supplemental data file is available in the Dryad Digital Repository: doi:10.5061/dryad.vg885.
(ZIP)

Dataset S3 **HuRef scaffolds localized using WCS data, along with their marginal localization probabilities for each chromosome. SD3_AllUnmappedScaffoldLocalizations.txt:** This file lists all unmapped scaffolds localized using WCS coverage data. Column 1 indicates the origin of each scaffold, either: 1_hg19 (an unmapped scaffold from the GRCh37 assembly), 2_BAC/Fos (a BAC or a Fosmid present in Genbank and included in the hs37d5 decoy sequences), 3_HuRefAssembly (a HuRef scaffold mapped to a chromosome in the HuRef assembly but not present in GRCh37, and included in the hs37d5 decoy sequences), 4_DeNovoCtg (a contig from the ALLPATHS-LG assembly of NA12878 included in the hs37d5 decoy sequences), 5_HuRefUnmapped (a HuRef unmapped scaffold/contig from the complete set of all HuRef assembly contigs). Scaffolds from “5_HuRefUnmapped” were localized using the complete HuRef assembly as the reference; scaffolds from all other categories were localized using hs37d5 as the reference. Column 2 indicates the GenBank ID or HuRef scaffold ID for each scaffold. Column 3 lists the chromosomal assignment of each scaffold in Genbank, if indicated (“NA” otherwise). Cases where GRCh37 and GRCh38 disagree are indicated, as are cases where the scaffold entry is listed as unmapped but the corresponding clone entry is localized (e.g. clones from libraries derived from a flow-sorted chr22 library). Column 4 indicates the chromosome assigned by Genovese et al. using admixture mapping, with the number of significant SNPs (LOD>6) indicated in brackets (figures are from an updated admixture mapping dataset received through personal correspondence); if no admixture map locus was determined, “NA” is listed. Column 5 lists the chromosomes assigned by our WCS coverage analysis (chromosomes with marginal localization probability estimates > = 0.9). Columns 6 to 29 list the marginal localization probability estimates for each chromosome given the available WCS data; a value of 1 indicates certainty under our Bayesian model that the scaffold localizes to that particular chromosome. This supplemental data file is available in the Dryad Digital Repository: doi:10.5061/dryad.vg885.
(ZIP)

Dataset S4 **Y haplogroup assignments and HSat3A6 array size estimates. SD4_HSat3A6ArraySizeEstimates.txt:** This file lists the 396 male samples for whom we estimated HSat3A6 array sizes. The first column lists the Sample ID, the second column lists the sample population (abbreviated according to the conventions used by the 1000 Genomes Project), the third and fourth columns specify the Y haplogroup assignment of each sample (from personal correspondence with Chris Tyler-Smith), and the fifth column lists our HSat3A6 array size estimate in bp. This supplemental data file is available in the Dryad Digital Repository: doi:10.5061/dryad.vg885.
(ZIP)
