# Supplementary figures and images for: Genomic Characterization of Large Heterochromatic Gaps in the Human Genome Assembly
Source: PLoS Comput Biol. 2014 May 15;10(5):e1003628. doi: 10.1371/journal.pcbi.1003628 (PMC4022460; doi:10.1371/journal.pcbi.1003628)

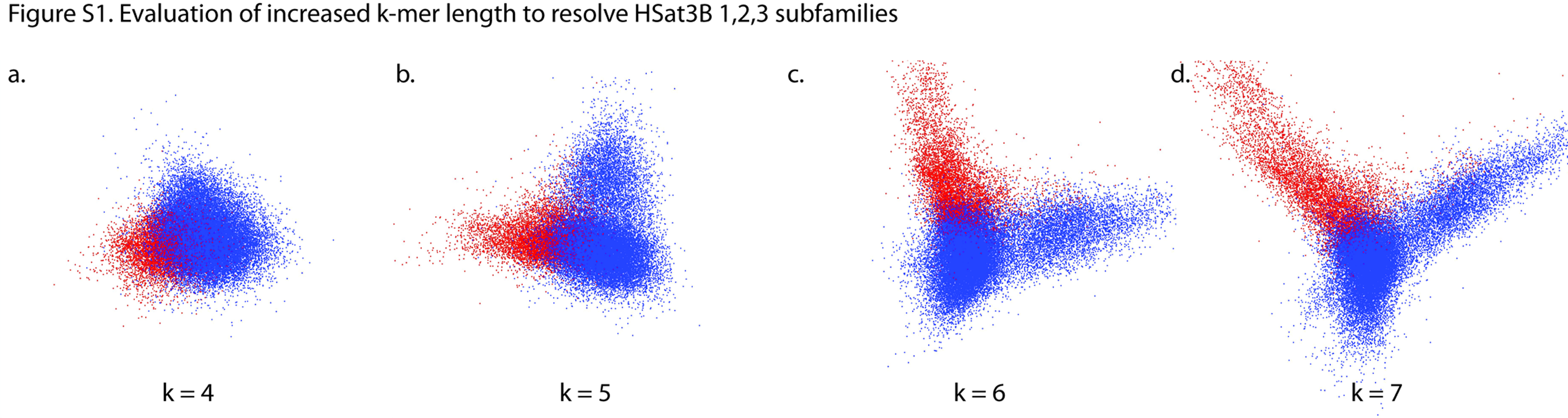

Supplement: Figure S1 — Evaluation of increased k -mer length to resolve HSat3B (1,2,3) subfamilies. Projections of HSat3B read frequency vectors (from the subgraph preceding subdivisions into HSat3B1, 2, and 3 subfamilies, referenced in Figure 2 in our main manuscript) are shown at varying lengths of k (where k = 4 (a), k = 5 (b), k = 6 (c), and k = 7 (d)). Reads previously assigned to HSat3B3 (with a 94.8% self-mate-pair frequency provided in our original study) are shown in blue, and the subsequent binary division, consisting of reads from HSat3B1 and 2 (with an 80.8% self-mate-pair frequency), are shown in red. (TIF) [file pcbi.1003628.s001.tif]

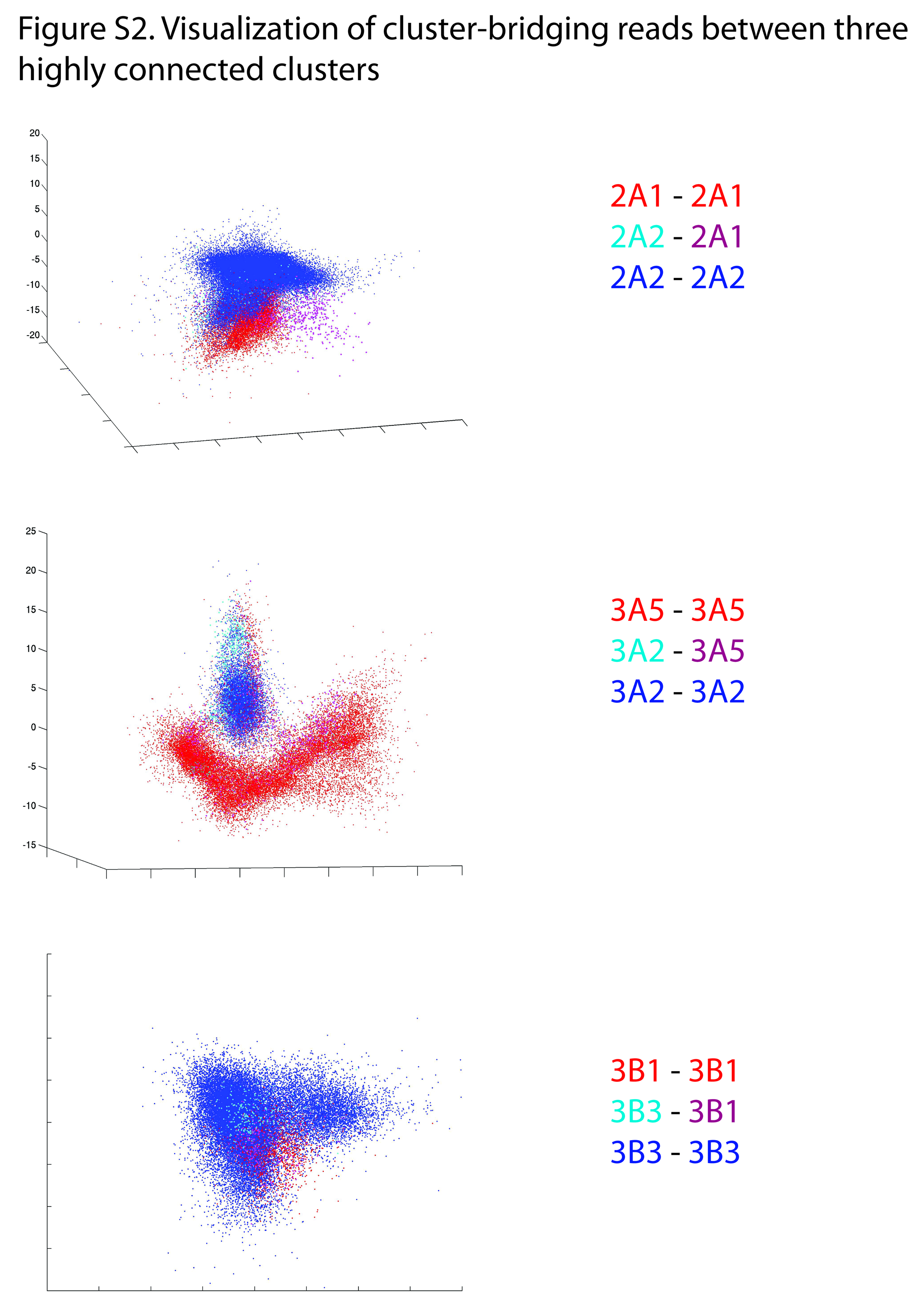

Supplement: Figure S2 — Visualization of cluster-bridging reads between 3 highly connected clusters. Top: 3D PCA projection (principal components 4,5,6) of HSat2A1 (red) and 2A2 (blue). 2A1 reads pairing to 2A2 are colored magenta, and their paired 2A1 reads are colored cyan. Middle: 3D PCA projection (principal components 1,2,3) of HSat3A5 (red) and 3A2 (blue). 3A5 reads pairing to 3A2 are colored magenta, and their paired 3A2 reads are colored cyan. Bottom: 2D PCA projection (principal components 1,2) of HSat3B1 (red) and 3B3 (blue). 3B1 reads pairing to 3B3 are colored magenta, and their paired 3B3 reads are colored cyan. For 3D plots, a perspective was selected to maximize the visible distinction of cluster-bridging reads. This distinction illustrates the fact that cluster-bridging reads may represent subregions of close homology between clusters. (TIF) [file pcbi.1003628.s002.tif]

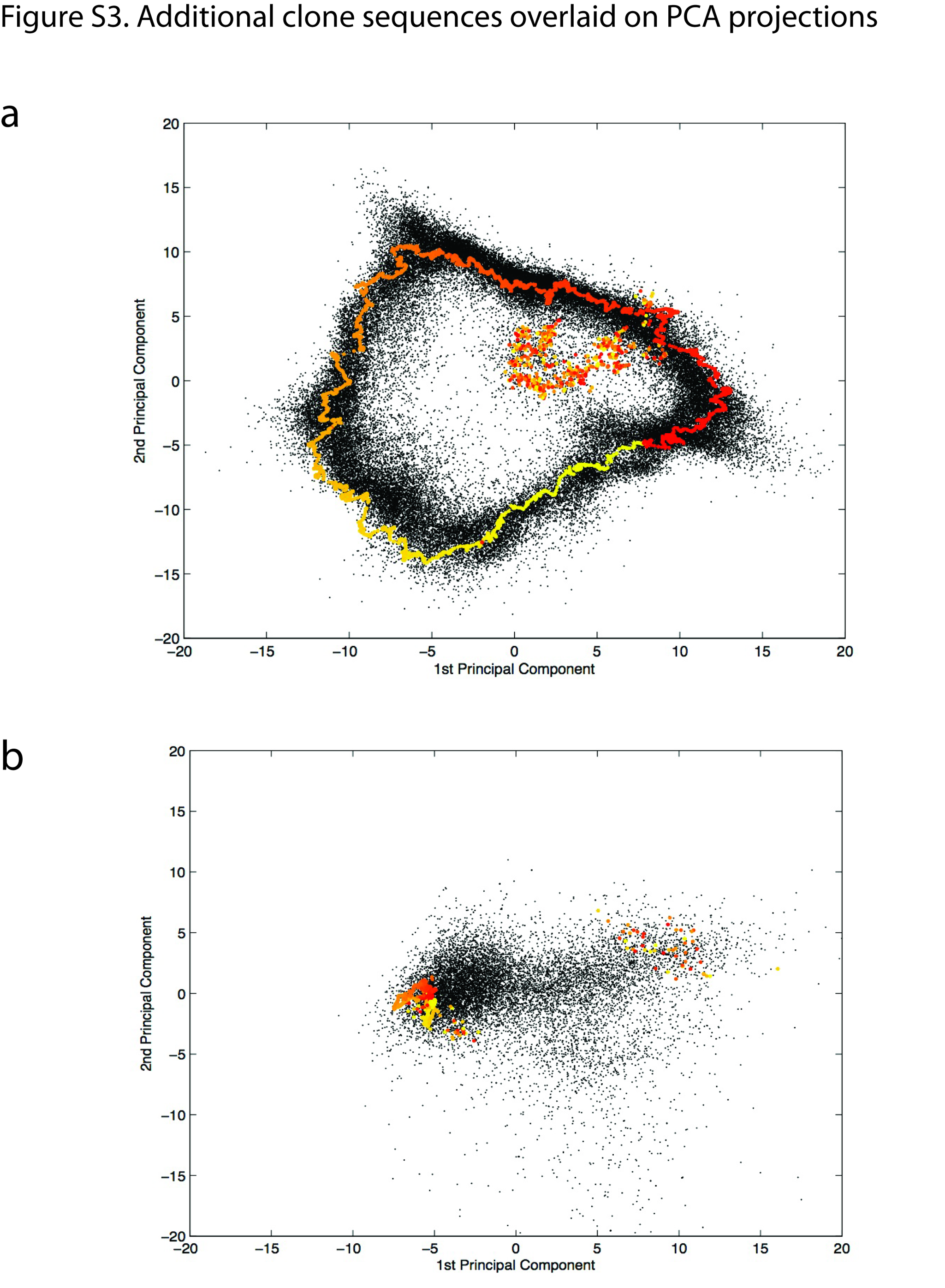

Supplement: Figure S3 — Additional clone sequences overlaid on PCA projections. Reads were simulated from complete clone sequences chrY DYZ1 (a) and pTRS-47 (b) and overlaid on PCA projections of HSat3A4 and HSat3A6, respectively (black points), colored by their start positions on each clone sequence. (TIF) [file pcbi.1003628.s003.tif]

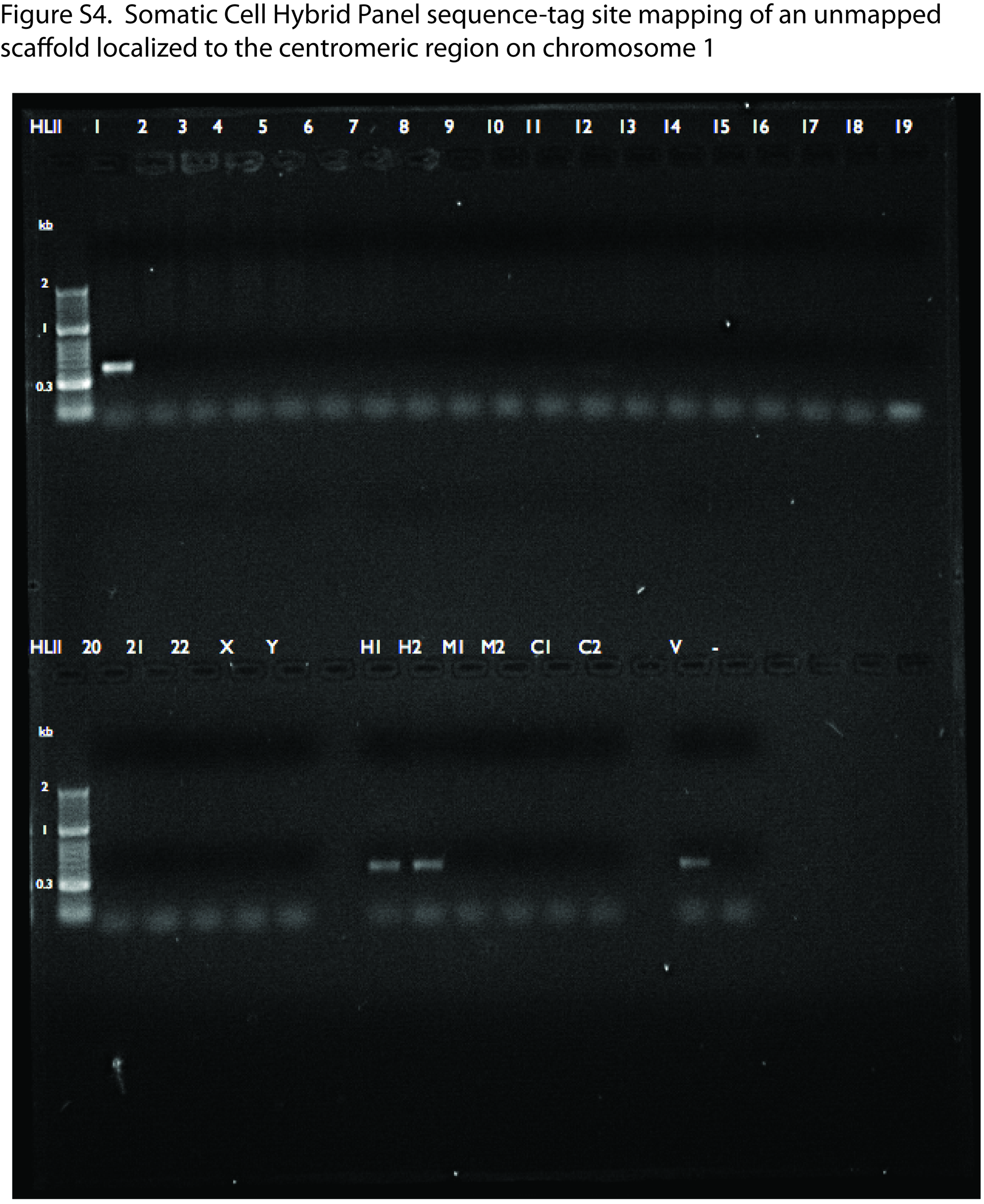

Supplement: Figure S4 — Somatic Cell Hybrid Panel Sequence-tag site mapping of unmapped scaffold localized to the centromeric region on chromosome 1. PCR was performed on a panel of DNA from human-rodent hybrid cells containing a single human chromosome each. We used a set of primers designed to be unique to SCAF_1103279187792 and demonstrate amplification only in the samples containing human chromosome 1. “HLII”: HyperLadderII; “1-24,X,Y”: rodent-human hybrid DNA samples containing each human chromosome in a rodent background; “H1,H2”: positive control whole-genome DNA from human donors; “M1,M2”: negative control whole-genome DNA from mouse background; “C1,C2”: negative control whole-genome DNA from Chinese hamster background; “V”: positive control whole-genome DNA from the HuRef donor individual; “-”: no DNA negative control. (TIF) [file pcbi.1003628.s004.tif]

Table S2. Proportion of self-paired reads in all pairwise subfamily comparisons.

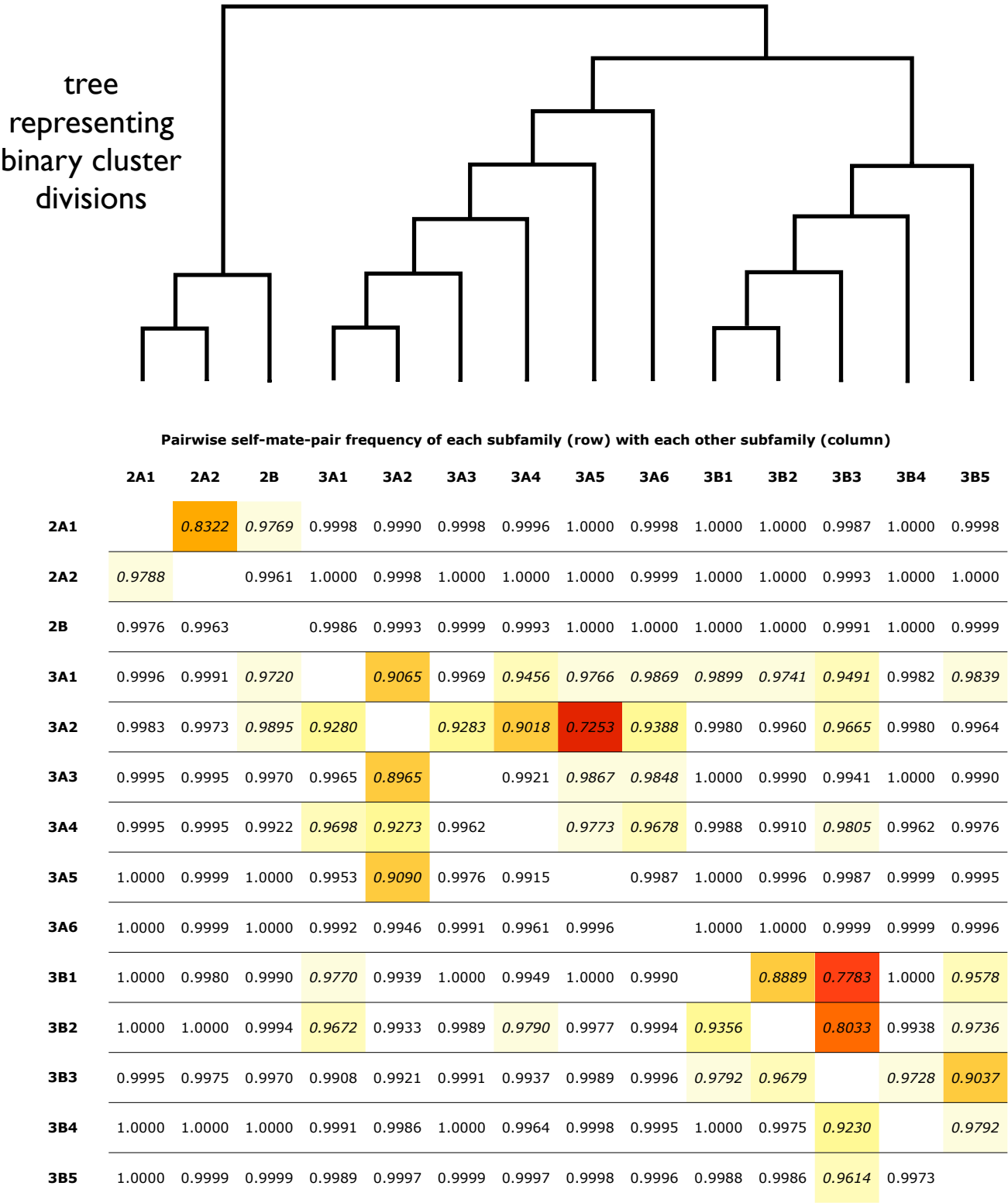

Supplement: Table S2 — Proportion of self-paired reads in all pairwise subfamily comparisons. Each self-mate-pair frequency value is calculated as (A→A)/(A→A +A→B), where A and B represent the two clusters being compared and A→B represents the number of mate pairs from A that belong in cluster B. Self-mate-pair frequencies are shaded according to their values, with lower values shaded red. Above: a tree illustrating the subgraph divisions used to generate the final fourteen clusters, as a point of comparison. (PDF) [file pcbi.1003628.s006.pdf]

**Table S3. Proportion of redundant 24-mer overlap in all pairwise subfamily comparisons.**

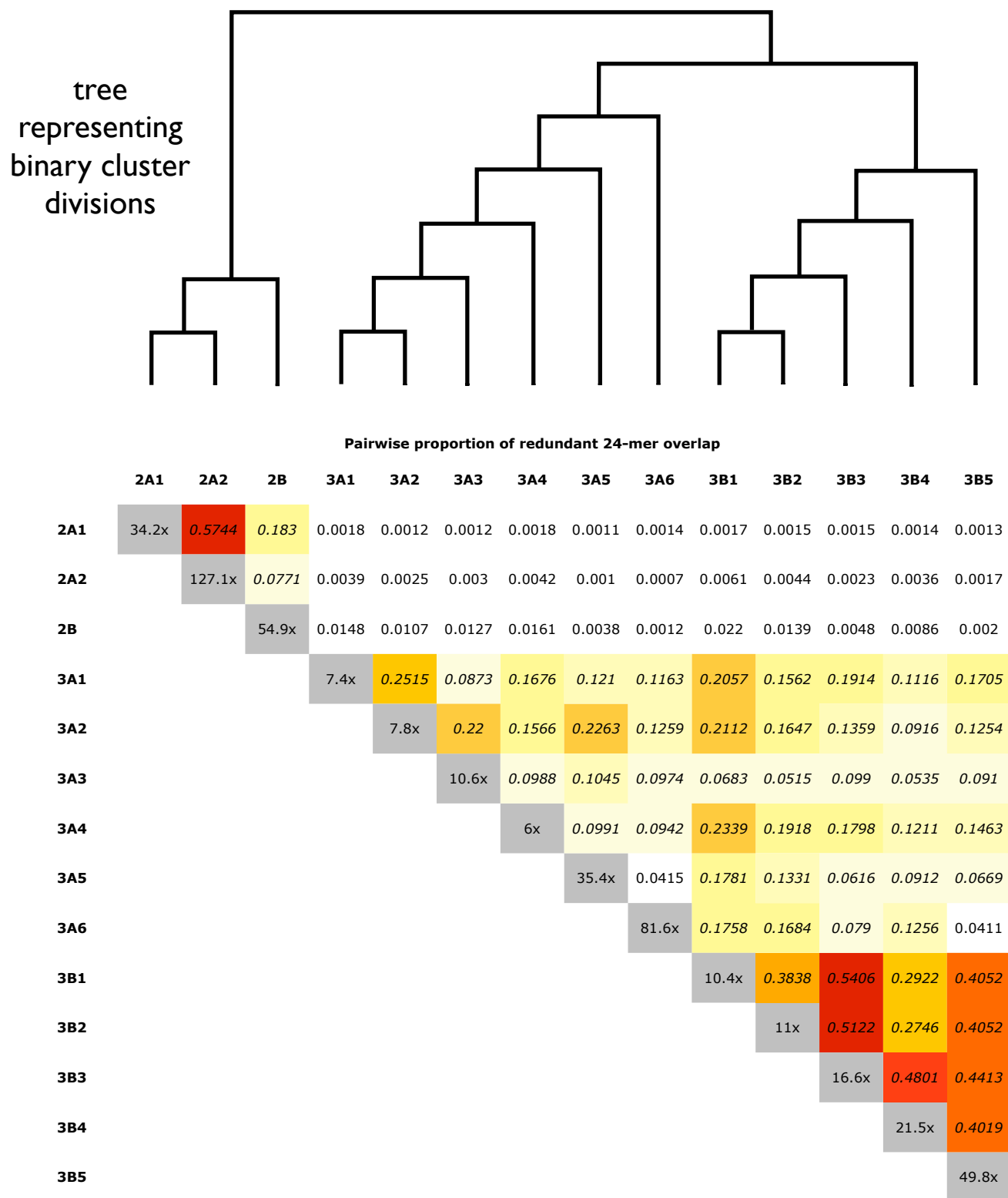

Supplement: Table S3 — Proportion of redundant 24-mer overlap in all pairwise subfamily comparisons. Each value represents the proportion of all redundant 24-mers in the smaller subfamily's reference database that are found in the larger subfamily's reference database (without replacing 24-mers in the larger database that have already been counted). Intuitively, this estimates the proportion of the smaller cluster that is ‘contained in’ the larger cluster. Values are colored yellow to red from low to high. Along the diagonal is the fold compression within each subfamily, which is the fold reduction in the number of 24-mers when redundant 24-mers are eliminated, a measure of each cluster's self-similarity. (PDF) [file pcbi.1003628.s007.pdf]
