# Supplementary material for: Genomic Characterization of Large Heterochromatic Gaps in the Human Genome Assembly
Source: PLoS Comput Biol. 2014 May 15;10(5):e1003628. doi: 10.1371/journal.pcbi.1003628 (PMC4022460; doi:10.1371/journal.pcbi.1003628)
Supplement: Table S1 — Comparisons of HSat2,3 subfamilies with published clone sequences. Each clone sequence was scanned for exact matches to subfamily-specific 24-mers from each HSat2,3 subfamily and assigned accordingly. (PDF) [file pcbi.1003628.s005.pdf]

Table S1. Comparisons of HSat2,3 subfamilies with published clone sequences

Subfamily Name	Published clone sequences matching subfamily-specific 24-mers [citation]	Published clone chromosomal localizations	Published repeat unit size (bp)
**HSat2A1**	cMEN375 [Jackson et al. 1992]	10	-
**HSat2A2**	pUC1.77 [Cooke and Hindley 1979]	1	1775
	cb2 [Jeanpierre et al. 1994]	1	-
**HSat2B**	pHuR195 [Moyzis et al. 1987]	16	-
	HUMPPD17 [Deininger et al. 1981]	-	-
**HSat3A1**	pW-1* [Bandyopadhyay et al. 2001]	13,21	1524
	cMEN219 [Jackson et al. 1993]	10	-
	QP23 [Jeanpierre et al. 1985]	22	-
**HSat3A2**	pE-1* [Bandyopadhyay et al. 2001]	13,14,21	1748
	pK-1* [Bandyopadhyay et al. 2001]	14,22	806
**HSat3A3**			
**HSat3A4**	pTRS-47* [Choo et al. 1990]	14,22	1366
	pR-1* [Bandyopadhyay et al. 2001]	13,14,15,21,22	1529
	pR-2* [Bandyopadhyay et al. 2001]	13,14,15,21,22	1556
	pR-4* [Bandyopadhyay et al. 2001]	13,14,15,21,22	1026
	pE-2* [Bandyopadhyay et al. 2001]	13,14,21	584
**HSat3A5**	HUMRSKPNI [Higgins et al. 1985]	15	1800/3600
**HSat3A6**	chrY-DYZ1* [Nakahori et al. 1986]	Y	3564
**HSat3B1**			
**HSat3B2**	pTRS-63* [Choo et al. 1992]	14	1404
	HUMASAT3* [Vissel et al. 1992]	13,14,21	1483
**HSat3B3**	HUMPPD9 [Deininger et al. 1981]	-	-
**HSat3B4**			
**HSat3B5**	pHuR98 [Moyzis et al. 1987]	9	-

*these clones represent complete repeat units that have been sequenced. All others are incomplete fragments.
