# Supplementary material for: Genomic Characterization of Large Heterochromatic Gaps in the Human Genome Assembly
Source: PLoS Comput Biol. 2014 May 15;10(5):e1003628. doi: 10.1371/journal.pcbi.1003628 (PMC4022460; doi:10.1371/journal.pcbi.1003628)
Supplement: Table S4 — Subfamily-specific 24-mers identified within each subfamily. Column 1 lists the number of high-quality (all bases have phred>20) 24-bp windows across all reads in each subfamily's read database (note: this is limited to 24-bp windows matching 24-mers seen on at least 2 reads in the entire HSat2,3 dataset, to reduce 24-mers likely to contain sequencing errors). Column 2 lists the total number of non-redundant 24-mers across all of these windows. Column 3 lists the “fold compression,” calculated as the quotient of Column 1 and Column 2, a measure of the self-similarity of the sequences in each cluster. Column 4 lists the number of 24-mers that are subfamily-specific (defined as present on >1% of reads in that subfamily and <0.1% of reads in any other subfamily, and on no non-HSat2,3 reads). The last column lists the proportion of 24-bp windows (from Column 1) that match a subfamily-specific 24-mer. (PDF) [file pcbi.1003628.s008.pdf]

**Table S4. Subfamily-specific 24-mers identified within each subfamily.**

| | All redundant 24-mers (>2x) | Non-redundant 24-mers | Fold compression | Non-redundant, subfamily-specific 24-mers | Prop. all 24-mers matching subfamily-specific 24-mer |
|---|---|---|---|---|---|
| **2A1** | 4944839 | 144609 | 34.2 | 1438 | 0.1839 |
| **2A2** | 36975281 | 290834 | 127.1 | 773 | 0.1019 |
| **2B** | 41324950 | 753334 | 54.9 | 6942 | 0.548 |
| **3A1** | 2983321 | 404377 | 7.4 | 446 | 0.0138 |
| **3A2** | 4839857 | 620427 | 7.8 | 756 | 0.0488 |
| **3A3** | 2028674 | 192076 | 10.6 | 6554 | 0.4499 |
| **3A4** | 4567620 | 767622 | 6.0 | 65 | 0.0024 |
| **3A5** | 10245675 | 289086 | 35.4 | 956 | 0.043 |
| **3A6** | 31322460 | 383824 | 81.6 | 2696 | 0.4593 |
| **3B1** | 1169330 | 111918 | 10.4 | 2689 | 0.2049 |
| **3B2** | 2121628 | 192363 | 11.0 | 3183 | 0.1545 |
| **3B3** | 12620891 | 760552 | 16.6 | 532 | 0.0178 |
| **3B4** | 3734728 | 173652 | 21.5 | 1238 | 0.1187 |
| **3B5** | 27294438 | 547585 | 49.8 | 945 | 0.1219 |
