# Supplementary material for: Genomic Characterization of Large Heterochromatic Gaps in the Human Genome Assembly
Source: PLoS Comput Biol. 2014 May 15;10(5):e1003628. doi: 10.1371/journal.pcbi.1003628 (PMC4022460; doi:10.1371/journal.pcbi.1003628)
Supplement: Table S5 — WCS datasets used in this analysis. Each row represents one of the 32 whole chromosome shotgun datasets used in this analysis, listing the target chromosome, the ID of the donor individual, the sex of the donor, the sequencing center (SC = Sanger Center, WUGSC = Washington University, WIBR = Broad Institute), the total number of Sanger sequencing reads, and the search terms required for downloading each dataset from the NCBI trace archive. (PDF) [file pcbi.1003628.s009.pdf]

Table S5. WCS datasets used in this analysis.

Target chr(s)	Donor ID	Donor sex	Center code	No. reads	Trace Archive query terms
1	11321	M	SC	606,874	center_project='CHR_1_11321'
1	17109	M	SC	737,140	center_project='CHR_1_17109'
1	07340	F	SC	679,330	center_project='CHR_1_7340'
2	17119	F	WUGSC	912,044	center_name = 'WUGSC' and species_code='HOMO SAPIENS' and (chromosome='CHR02' or chromosome='2') and strategy='WCS'
4	17119	F	WIBR	619,329	center_project='S246'
4-5	17119	F	WIBR	363,729	center_project='S223'
5	17119	F	WIBR	480,263	center_project='S224' or center_project = 'S247'
6	11321	M	SC	561,174	center_project='CHR_6_11321'
6	17119	F	SC	506,469	center_project='CHR_6_17119'
6	07340	F	SC	568,268	center_project='CHR_6_7340'
7	17119	F	WUGSC	311,439	strategy = 'WCS' and center_name = 'WUGSC' and chromosome='7'
8-9	17119	F	WIBR	685,493	center_project='S225' or center_project='S248'
9-12	07340	F	SC	938,978	center_project='CHR_9-12_7340'
13	11321	M	SC	318,804	center_project='CHR_13_11321'
13	17119	F	SC	317,029	center_project='CHR_13_17119'
13	07340	F	SC	348,128	center_project='CHR_13_7340'
14	17119	F	WIBR	353,264	center_project='S226' or center_project='S249'
18	17109	M	WIBR	276,943	center_project='S227' or center_project='S250'
20	10470	M	SC	287,810	seq_lib_id='45079'
20	11321	M	SC	371,611	seq_lib_id='47841' or seq_lib_id='45081'
20	17119	M	SC	264,442	seq_lib_id='45080'
20	07340	F	SC	361,607	seq_lib_id='44340'
21	17119	F	WUGSC	116,714	strategy='WCS' and center_name='WUGSC' and chromosome='21'
22	10470	M	SC	220,600	center_project='CHR_22_10470'
22	11321	M	SC	265,034	center_project='CHR_22_11321'
22	17119	F	SC	202,925	center_project='CHR_22_17119'
22	07340	F	SC	222,795	center_project='CHR_22_7340'
X	10470	M	SC	48,419	center_project='CHR_X_10470'
X	11321	M	SC	394,822	center_project='CHR_X_11321'
X	17119	F	SC	463,386	center_project='CHR_X_17119'
X	07340	F	SC	463,719	center_project='CHR_X_7340'
Y	17109	M	WIBR	96,768	center_project='S228'
