# Supplementary material for: Genomic Characterization of Large Heterochromatic Gaps in the Human Genome Assembly
Source: PLoS Comput Biol. 2014 May 15;10(5):e1003628. doi: 10.1371/journal.pcbi.1003628 (PMC4022460; doi:10.1371/journal.pcbi.1003628)
Supplement: Table S6 — Mixing matrix estimating percent of WCS reads from each chromosome for each sample. Each row represents one WCS dataset and lists the estimated percentage of reads from each chromosome based on coverage of unique 24-mers on each chromosome. The last row gives the relative sizes of each chromosome for comparison. Column labels shaded light grey correspond to chromosomes present only in mixtures with other chromosomes, and those shaded dark grey are the 5 chromosomes untargeted by any of the available datasets. Percentages greater than 75 are shaded yellow; those between 20 and 75 are shaded orange and correspond to mixed chromosome datasets. (PDF) [file pcbi.1003628.s010.pdf]

Table S6. Mixing matrix estimating percent of WCS reads from each chromosome for each sample.

Target Chr(s)	Donor ID	1	2	3	4	5	6	7	8	9	10	11	12	13	14	15	16	17	18	19	20	21	22	X	Y
1	11321	85.13	1.92	1.05	0.15	1.30	2.29	0.70	0.94	0.61	0.50	0.40	0.42	0.31	0.69	0.76	0.77	0.56	0.22	0.21	0.30	0.19	0.25	0.28	0.05
1	17109	78.63	1.44	1.50	0.39	1.88	1.71	2.19	0.90	1.04	0.99	0.85	1.02	0.81	1.03	1.27	1.02	0.69	0.27	0.37	0.45	0.28	0.37	0.69	0.20
1	7340	79.89	0.56	0.49	0.11	1.78	1.53	1.67	1.23	0.81	0.96	1.00	0.74	0.27	1.65	1.51	1.26	1.00	0.23	0.59	0.30	0.37	0.28	1.78	0.00
2	17119	3.88	74.44	1.32	1.59	1.76	1.26	1.59	1.29	1.06	0.91	0.83	1.05	2.61	0.64	1.00	0.35	0.11	1.25	0.15	0.56	0.40	0.30	1.63	0.00
4-5	17119	0.33	0.38	1.45	71.42	22.28	1.13	0.24	0.20	0.20	0.17	0.18	0.21	0.37	0.21	0.16	0.12	0.10	0.29	0.05	0.07	0.07	0.08	0.30	0.00
4	17119	0.30	0.31	1.25	94.99	0.33	0.28	0.22	0.23	0.20	0.19	0.17	0.18	0.27	0.13	0.13	0.09	0.08	0.16	0.04	0.08	0.06	0.07	0.21	0.00
5	17119	0.49	0.53	2.06	2.01	86.63	3.58	0.35	0.40	0.27	0.25	0.26	0.28	0.74	0.35	0.25	0.17	0.13	0.47	0.07	0.10	0.13	0.07	0.42	0.00
6	11321	0.30	0.34	0.28	0.31	3.02	92.01	0.29	0.24	0.23	0.20	0.21	0.24	0.48	0.21	0.23	0.15	0.12	0.24	0.05	0.14	0.12	0.07	0.42	0.11
6	17119	0.37	0.40	0.49	0.46	9.40	82.62	0.46	0.35	0.28	0.31	0.24	0.29	1.26	0.22	0.29	0.13	0.20	0.58	0.08	0.20	0.36	0.06	0.97	0.01
6	7340	0.33	0.33	0.32	0.29	5.49	88.24	0.23	0.20	0.18	0.18	0.18	0.20	2.21	0.27	0.17	0.09	0.09	0.25	0.05	0.17	0.13	0.07	0.30	0.01
7	17119	0.51	0.65	0.59	0.43	0.53	1.29	87.78	1.01	0.61	0.34	0.32	0.43	1.57	0.96	0.39	0.22	0.15	0.27	0.11	0.20	0.48	0.45	0.70	0.02
8-9	17119	0.52	0.62	0.56	0.50	0.55	0.59	0.87	59.43	26.51	0.35	0.28	0.96	0.44	0.49	0.42	0.18	0.13	0.47	0.09	0.29	0.37	0.09	5.27	0.00
9-12	7340	0.91	0.43	0.29	0.27	0.32	0.29	0.29	0.34	25.09	23.99	20.82	24.68	0.19	0.19	0.21	0.17	0.11	0.52	0.06	0.13	0.21	0.20	0.27	0.01
13	11321	0.48	0.53	0.48	0.73	0.45	0.35	0.33	0.33	0.56	0.46	0.38	0.48	92.71	0.24	0.20	0.15	0.15	0.21	0.12	0.13	0.13	0.11	0.16	0.14
13	17119	0.45	0.52	0.49	0.39	0.34	0.36	0.34	0.33	0.21	0.28	0.27	0.33	93.04	0.41	0.42	0.23	0.20	0.33	0.14	0.17	0.18	0.17	0.40	0.00
13	7340	0.23	0.25	0.20	0.43	0.20	0.26	0.16	0.18	0.19	0.15	0.16	0.16	96.50	0.12	0.10	0.10	0.08	0.10	0.04	0.09	0.06	0.06	0.18	0.00
14	17119	1.20	1.24	0.94	0.76	0.76	0.70	0.89	0.63	0.67	0.69	0.69	0.79	0.58	83.16	3.10	0.46	0.33	0.34	0.22	0.51	0.50	0.23	0.59	0.00
18	17109	1.01	1.01	0.73	0.75	0.71	1.28	0.86	0.68	0.60	0.58	0.61	0.51	0.52	0.68	0.44	0.34	0.56	86.24	0.22	0.26	0.24	0.16	0.56	0.45
20	10470	0.41	0.39	0.32	0.30	0.34	0.29	0.28	0.23	0.32	0.38	0.22	0.21	0.19	0.20	0.22	0.21	0.32	0.16	0.16	94.28	0.14	0.12	0.16	0.14
20	11321	0.60	0.54	0.43	0.45	0.42	0.39	0.36	0.34	0.33	0.40	0.33	0.29	0.24	0.25	0.25	0.29	0.36	0.19	0.30	92.47	0.15	0.37	0.11	0.13
20	17119	0.86	0.82	0.68	0.58	0.53	0.60	0.46	0.49	0.48	0.55	0.43	0.51	0.38	0.36	0.31	0.32	0.36	0.30	0.28	89.24	0.25	0.73	0.50	0.00
20	7340	0.42	0.37	0.29	0.28	0.28	0.26	0.19	0.22	0.23	0.20	0.22	0.23	0.15	0.14	0.14	0.20	0.21	0.13	0.12	95.32	0.08	0.14	0.17	0.00
21	17119	1.69	1.86	1.46	1.12	1.26	1.11	1.07	1.25	0.88	0.97	0.95	0.97	0.77	1.05	1.23	0.65	0.44	0.68	0.33	0.45	78.48	0.33	0.96	0.03
22	10470	0.56	0.44	0.40	0.36	0.33	0.34	0.34	0.32	0.32	0.25	0.28	0.27	0.25	0.23	0.38	0.22	0.28	0.16	0.13	0.20	0.12	93.61	0.13	0.06
22	11321	0.56	0.65	0.52	0.43	0.52	0.48	0.42	0.36	0.32	0.34	0.35	0.35	0.39	0.27	0.27	0.35	0.38	0.18	0.24	0.25	0.23	90.47	1.57	0.10
22	17119	0.79	0.70	0.67	0.64	0.55	0.56	0.50	0.43	0.38	0.42	0.40	0.38	0.39	0.36	0.34	0.32	0.32	0.22	0.16	0.29	0.21	90.49	0.47	0.00
22	7340	0.86	0.85	0.65	0.68	0.67	0.72	0.54	0.59	0.57	0.52	0.49	0.45	0.48	0.44	0.36	0.33	0.52	0.28	0.26	0.24	0.14	88.86	0.47	0.01
X	10470	0.20	0.33	0.20	0.20	0.26	0.20	0.94	2.61	0.19	0.25	0.18	0.28	2.39	0.34	0.25	0.16	0.08	0.97	0.07	0.14	0.83	0.05	88.89	0.00
X	11321	0.23	0.32	0.27	0.19	0.25	0.33	2.28	2.35	0.28	0.32	0.31	0.33	4.07	0.26	0.20	0.18	0.15	0.43	0.09	0.12	1.60	0.12	85.19	0.13
X	17119	0.48	0.72	0.39	0.35	0.37	0.38	1.49	1.38	0.32	0.34	0.30	0.37	1.52	0.25	0.29	0.20	0.17	0.27	0.11	0.17	0.57	0.09	89.44	0.03
X	7340	0.24	0.66	0.18	0.13	0.19	0.17	1.42	1.32	0.15	0.20	0.18	0.19	1.66	0.20	0.12	0.11	0.10	0.27	0.07	0.07	0.60	0.07	91.70	0.00
Y	17109	0.93	0.75	0.67	0.82	0.70	0.69	0.43	0.42	0.55	0.51	0.47	0.51	0.41	0.36	0.40	0.27	0.22	0.35	0.17	0.21	0.19	0.13	0.18	89.67
ALL	hg19	8.34	8.14	6.63	6.40	6.05	5.73	5.33	4.90	4.73	4.54	4.52	4.48	3.85	3.59	3.43	3.02	2.72	2.61	1.98	2.11	1.61	1.72	2.60	0.99
