# Supplementary material for: Genomic Characterization of Large Heterochromatic Gaps in the Human Genome Assembly
Source: PLoS Comput Biol. 2014 May 15;10(5):e1003628. doi: 10.1371/journal.pcbi.1003628 (PMC4022460; doi:10.1371/journal.pcbi.1003628)
Supplement: Table S7 — HSat2,3 localization predictions from WCS data. For each subfamily and each chromosome, this table lists the median abundance estimate (in kb) of 3,000 samples from the posterior distribution along with the boundaries of a 95% highest posterior density interval. Confident estimates above 50 kb are colored according to size, yellow being smallest and red being largest. Shaded grey are two potentially artifactual results inconsistent with hybridization-based studies for the localization of these subfamilies. Row labels shaded light grey correspond to chromosomes present only in mixtures with other chromosomes, and those shaded dark grey are the 5 chromosomes untargeted by any of the available datasets. Some of these untargeted chromosomes still obtain confident abundance estimates given information inferred from their varying contamination of WCS datasets targeting other chromosomes. (PDF) [file pcbi.1003628.s011.pdf]

Table S7. HSat2,3 localization predictions from WCS data.

	2A1	2A2	2B	3A1	3A2	3A3	3A4	3A5	3A6	3B1	3B2	3B3	3B4	3B5
1	1474 [1447,1499]	14270 [14195,14349]	202 [159,244]	44 [39,51]	27 [21,34]	0 [0,1]	26 [17,34]	28 [14,43]	1 [0,2]	9 [6,11]	245 [235,256]	111 [98,125]	5 [1,8]	32 [19,43]
2	2 [0,5]	48 [19,75]	523 [495,551]	2 [0,6]	8 [2,13]	0 [0,1]	9 [2,16]	7 [0,18]	0 [0,1]	1 [0,2]	0 [0,1]	4 [0,11]	4 [0,8]	17 [2,32]
3	7 [0,23]	10 [0,33]	120 [0,335]	13 [0,43]	19 [0,64]	4 [0,14]	27 [0,89]	27 [0,84]	7 [0,22]	5 [0,15]	6 [0,20]	165 [0,461]	9 [0,32]	46 [0,150]
4	0 [0,1]	0 [0,1]	1 [0,3]	0 [0,1]	2 [0,4]	0 [0,1]	23 [18,27]	2 [0,4]	1 [0,2]	0 [0,1]	0 [0,1]	14 [8,20]	0 [0,1]	1 [0,3]
5	1 [0,3]	1 [0,2]	15 [5,25]	1 [0,3]	1 [0,2]	0 [0,1]	1 [0,3]	1 [0,3]	0 [0,1]	0 [0,1]	1 [0,2]	149 [133,164]	1 [0,2]	43 [32,54]
6	1 [0,2]	1 [0,2]	6 [2,9]	0 [0,1]	1 [0,2]	0 [0,0]	0 [0,1]	3 [1,6]	1 [0,2]	0 [0,1]	1 [0,2]	5 [1,8]	1 [0,3]	1 [0,4]
7	1 [0,4]	4 [0,11]	347 [321,373]	4 [0,8]	17 [11,23]	6 [3,10]	7 [1,13]	4 [0,8]	94 [82,107]	1 [0,3]	1 [0,3]	158 [141,176]	7 [2,12]	31 [19,46]
8	1 [0,3]	1 [0,2]	22 [4,38]	1 [0,2]	1 [0,2]	0 [0,1]	4 [0,7]	1 [0,4]	0 [0,1]	0 [0,1]	1 [0,2]	39 [0,76]	1 [0,3]	1225 [1132,1325]
9	2 [0,6]	2 [0,5]	25 [0,59]	1 [0,4]	1 [0,4]	0 [0,1]	5 [0,11]	1 [0,5]	1 [0,2]	0 [0,1]	1 [0,2]	109 [14,188]	1 [0,4]	7260 [7100,7421]
10	134 [0,298]	62 [0,131]	26 [0,68]	21 [0,47]	24 [0,55]	1 [0,2]	6 [0,16]	3 [0,8]	3 [0,8]	1 [0,3]	1 [0,2]	68 [0,174]	2 [0,6]	30 [0,100]
11	202 [1,368]	47 [0,124]	28 [0,73]	23 [0,52]	27 [0,62]	1 [0,2]	7 [0,19]	3 [0,9]	3 [0,9]	1 [0,3]	1 [0,3]	62 [0,168]	3 [0,8]	32 [0,109]
12	56 [0,168]	43 [0,108]	26 [0,66]	17 [0,41]	16 [0,43]	0 [0,2]	6 [0,16]	3 [0,8]	3 [0,7]	1 [0,2]	1 [0,2]	76 [0,177]	2 [0,6]	32 [0,106]
13	4 [3,6]	2 [0,4]	9 [6,13]	85 [80,91]	49 [45,53]	1 [0,2]	152 [145,160]	8 [5,10]	4 [2,6]	2 [1,3]	15 [13,18]	159 [151,167]	89 [83,95]	52 [47,57]
14	1 [0,2]	1 [0,2]	3 [0,8]	29 [23,35]	59 [50,68]	1 [0,2]	120 [108,133]	26 [7,45]	4 [1,6]	1 [0,2]	1 [0,3]	231 [214,249]	101 [91,112]	109 [95,124]
15	6 [0,21]	7 [0,26]	446 [92,803]	20 [0,64]	35 [0,109]	3 [0,10]	51 [0,157]	1308 [812,1778]	5 [0,18]	5 [0,16]	6 [0,19]	77 [0,241]	20 [0,64]	50 [0,175]
16	14 [0,48]	15 [0,50]	4436 [2088,6450]	27 [0,93]	46 [0,145]	4 [0,14]	67 [0,208]	425 [0,1039]	9 [0,31]	10 [0,34]	12 [0,40]	107 [0,338]	24 [0,77]	58 [0,193]
17	14 [0,46]	13 [0,46]	552 [2,1476]	27 [0,88]	50 [0,155]	5 [0,16]	53 [0,166]	157 [0,464]	10 [0,32]	10 [0,32]	17 [0,56]	62 [0,206]	21 [0,71]	42 [0,136]
18	0 [0,1]	1 [0,3]	1 [0,4]	0 [0,1]	0 [0,1]	0 [0,1]	0 [0,1]	2 [0,4]	0 [0,1]	0 [0,1]	0 [0,1]	0 [0,1]	0 [0,1]	0 [0,1]
19	16 [0,56]	15 [0,53]	931 [0,2313]	38 [0,124]	74 [0,229]	6 [0,20]	145 [0,392]	265 [0,720]	11 [0,39]	15 [0,51]	17 [0,59]	136 [0,427]	31 [0,98]	67 [0,218]
20	0 [0,0]	0 [0,0]	1 [0,2]	17 [16,20]	4 [3,5]	0 [0,1]	10 [8,12]	1 [0,2]	0 [0,1]	1 [0,1]	24 [22,26]	416 [407,425]	6 [5,8]	118 [113,123]
21	1 [0,3]	2 [0,6]	28 [17,39]	64 [54,74]	58 [48,67]	0 [0,1]	116 [103,129]	10 [5,17]	1 [0,2]	2 [1,4]	53 [45,62]	144 [129,159]	97 [85,110]	44 [34,56]
22	2 [1,3]	2 [0,4]	146 [140,152]	75 [71,79]	56 [52,59]	1 [0,1]	196 [189,202]	1 [0,2]	5 [4,7]	1 [1,2]	5 [4,6]	252 [244,259]	169 [164,176]	107 [102,113]
X	0 [0,1]	0 [0,1]	10 [5,14]	1 [0,2]	1 [0,2]	1 [0,2]	6 [3,9]	2 [0,3]	1 [0,3]	0 [0,0]	0 [0,1]	2 [0,4]	4 [1,6]	0 [0,2]
Y	1 [0,2]	4 [0,10]	31 [21,42]	4 [1,8]	68 [56,80]	372 [343,399]	187 [168,210]	7 [2,12]	8235 [8110,8354]	1 [0,3]	1 [0,3]	22 [15,30]	3 [0,6]	42 [30,54]
