# Supplementary material for: Genomic Characterization of Large Heterochromatic Gaps in the Human Genome Assembly
Source: PLoS Comput Biol. 2014 May 15;10(5):e1003628. doi: 10.1371/journal.pcbi.1003628 (PMC4022460; doi:10.1371/journal.pcbi.1003628)
Supplement: Table S8 — HSat2,3 annotated on hg19 assembly fragments. This table lists all clones in the hg19 assembly that contain at least 1 kb of HSat2,3 as annotated by RepeatMasker. The fourth column designates whether each clone is centromere/heterochromatin proximal (within 500 kb) or located on the chromosome arms. The last column indicates the HSat2,3 subfamily assignment of each region based on perfect matches to subfamily-specific 24-mers. (PDF) [file pcbi.1003628.s012.pdf]

Table S8. HSat2,3 annotated on hg19 assembly fragments

Assembly Clone Name	Position (hg19)	HSat2,3 bp by RepeatMasker	arm/cen	HSat2,3 subfamilies assigned
RP11-417J8	1:142535434-142731022	4224	cen	3A4
CH17-158B1	2:89830436-90041857	45615	cen	2B,3A1
CH17-132F21	2:90371525-90545103	22157	cen	2B
RP11-575H3	2:91595103-91790098	20157	cen	2B
CTD-2053H7	2:92244957-92326171	5001	cen	2B
RP11-1281K21	4:49076738-49283503	61324	cen	3B3
RP11-241F15	4:49488941-49660117	25224	cen	3B3
RP11-1324A7	7:57518331-57586048	7958	cen	2B,3A4
XXFOS-8589D1	7:61054331-61083324	1496	cen	2B
RP11-715L17	7:61727020-61917157	41957	cen	2B
RP11-291L22	10:38652100-38818835	43709	cen	3A1
RP11-453N3	10:38868835-39075027	19185	cen	2B,3A1
RP11-96F8	10:39075027-39154935	72654	cen	3A2
RP11-745D9	10:42354935-42546687	40427	cen	2A1,3B3
XX-Y237C10	10:42596687-42794387	15048	cen	2B, 3A1
RP11-313J2	10:42794387-42879686	21673	cen	3A1
CTD-2522B17	16:33859975-34023150	32034	cen	2B,3A4
CTD-2144E22	16:34173150-34341696	16042	cen	2B
RP11-696P19	16:46385801-46508594	71251	cen	2B
RP11-260A9	17:25263006-25441564	3349	cen	3A4
RP5-854E16	20:29803908-29874360	27468	cen	3A1
bP-21264C1	21:10084920-10215976	1816	cen	3A4
CTD-2503J9	21:10697896-10906316	78276	cen	3A1,3A4
c20H12	22:16847850-16884805	14280	cen	2B
RP11-1126J10	Y:13104553-13143954	3840	cen	3A4
RP1-85D24	Y:13193954-13278549	1234	cen	3A4
RP11-886I11	Y:13401114-13543063	9403	cen	3A4
RP11-295P22	Y:13543063-13748578	94044	cen	3A3
RP11-75F5	Y:13798578-13958100	64991	cen	3A2
RP11-1136L22	Y:28785151-28819361	19233	cen	3A4
RP11-242E13	Y:58819361-58917656	90966	cen	3A6
RP11-57J19	Y:58967656-59034049	7270	cen	3A4
RP11-206L10	1:632917-812484	2807	arm	3A1
RP11-744H18	1:149004460-149110998	2617	arm	2B
RP11-504P24	1:224085051-224269695	4619	arm	3A1
RP11-667F9	7:64876309-65008648	1145	arm	2B,3A4
RP11-383B4	10:18804301-18926872	19712	arm	-
