# Supplementary material for: Genomic Characterization of Large Heterochromatic Gaps in the Human Genome Assembly
Source: PLoS Comput Biol. 2014 May 15;10(5):e1003628. doi: 10.1371/journal.pcbi.1003628 (PMC4022460; doi:10.1371/journal.pcbi.1003628)
Supplement: Table S9 — Large HSat2,3-associated unmapped scaffolds localized by WCS coverage. For each of the seventeen HSat2,3-associated large unmapped scaffolds, this table lists the total non-gap, non-RepeatMasked length (column 3); the number of unique 24-mers used for WCS mapping (column 4); the percentage of non-RepeatMasked bases in continuous stretches >500 bp that align to hg19 at >90% ID and >500 bp (column 5); the names of genes and pseudogenes (labeled (p)) annotated in GenBank (column 6); the chromosomes assigned by WCS coverage mapping in this study (column 7); the chromosomal bands assigned by Genovese et al. 2013 where applicable (column 8); the primary and secondary chromosomal bands assigned by fosmid FISH by Levy et al. 2007 (column 9); the number of HSat2,3 reads used to assemble the scaffold, by subfamily (column 10); the number of HSat2,3 reads paired with reads used to assemble the scaffold, by subfamily (column 11). (PDF) [file pcbi.1003628.s013.pdf]

Table S9 (page 1 of 2). Large HSat2,3-associated unmapped scaffolds localized by WCS coverage.

HuRef SCAF ID NCBI ref. ID	Non-gap length (bp)	Non-gap non-repeat length (bp)	Unique 24-mer count	% seg dup	NCBI gene predictions	Chr(s) assigned by WCS mapping	Genovese admixture map loci [#SNPs]	Levy FISH map loci PRIMARY/ SECONDARY	HSat2,3 read subfams [read ct.]	HSat2,3 mate pair subfams [read ct.]
**1103279188373 NW_001841157.1**	1,500,749	610,252	446,252	89.2	none	**X,Y**	X [2]	NA	2A2[8]	2A2[4] 3B5[1]
**1103279188189 NW_001841137.1**	270,611	113,266	30,200	95	MIR3118-3 (p)SNX18P16 (p)LOC644339 (p)LOC100129224 (p)LOC101060847 (p)FLJ43315	**9**	NA	13cen 22cen 9q13 / 14cen 15cen 21cen 1q12 4p12	NONE	3A4[9]
**1103279181222 NW_001839651.1**	236,390	93,415	20,586	86.3	LOC101060792 (p)LOC100291626 (p)LOC100287702 (p)LOC100996901 (p)LOC100134412 (p)LOC101060784	**3,14,16, 17,19**	NA	2cen 14cen 22cen 16cen / 9q21 9cen 15cen 13cen 10cen 1q11	2B[27] 3A4[3] 2A2[1]	2B[22] 3A4[3] 3A1[2]
**1103279188307 NW_001841150.1**	168,878	64,308	15,673	91.4	LOC101060770 (p)LOC101059996	**1**	1 [53]	1q11 16q11.2 7cen / 2p11.2 3p21.3 17p11	2B[250] 2A1[80] 2A2[57] 3B1[2] 3A4[1]	2B[43] 2A1[4] 2A2[1] 3B1[1] 3A4[1]
**1103279188429 NW_001841160.1**	172,513	59,515	14,687	84.1	(p)LOC101059923 (p)LOC101060813	**7**	7 [128]	1q12 7cen 16q11 / 2cen 9cen 13cen 14cen 15cen 22cen	2B[312] 3B3[124] 3B1[11] 2A2[11] 3B2[10] 3B5[7] 2A1[3] 3B4[1] 3A2[1]	2B[44] 3B3[22] 3B1[2] 3B5[2] 2A2[1] 3A2[1] 3A5[1]
**1103279187452 NW_001840853.1**	318,231	49,935	12,054	90.7	(p)LOC100996808	**20**	20 [563]	20q11 / 9qh 13cen 14cen 15cen 21cen 22cen	3A4[14]	3A4[15] 3B3[1]
**1103279188266 NW_001841145.1**	150,210	46,868	18,917	50.8	DUX4L4 LOC100292669 (p)LOC100653165 (p)DUX4L11 (p)LOC100653184 (p)LOC101060749 (p)LOC100653190 (p)LOC100288523 (p)LOC649425 (p)LOC100134340	**14,19**	NA	NA	3A4[5]	3A4[5]
**1103279179615 NW_001839257.1**	223,057	43,410	10,607	84.2	(p)LOC647654	**20**	20 [90]	NA	3A4[6]	3A4[5]

Table S9 (page 2 of 2). Large HSat2,3-associated unmapped scaffolds localized by WCS coverage.

HuRef SCAF ID NCBI ref. ID	Non-gap length (bp)	Non-gap non-repeat length (bp)	Unique 24-mer count	% seg dup	NCBI gene predictions	Chr(s) assigned by WCS mapping	Genovese admixture map loci [#SNPs]	Levy FISH map loci PRIMARY/ SECONDARY	HSat2,3 read subfams [read ct.]	HSat2,3 mate pair subfams [read ct.]
**1103279188305 NW_001841149.1**	210,648	40,192	6,298	79.8	none	**9**	2 [6]<br>9 [2]	NA	2B[282]<br>2A1[10]<br>2A2[9]	2B[53]<br>2A1[4]
**1103279187792 NW_001841011.1**	247,273	35,200	7,692	79.6	LOC100132859<br>LOC100505781<br>(p)LOC101060790	**1**	1 [197]	1q11 16q21 /<br>2p12 7q12	2B[247]<br>2A2[66]<br>2A1[45]	2B[50]<br>2A1[7]<br>2A2[6]
**1103279187616 NW_001840933.1**	48,268	28,883	8,249	88.5	none	**14,22**	22 [1]	NA	3A4[11]	3A4[11]
**1103279188290 NW_001841146.1**	127,632	26,401	5,299	72.9	(p)LOC100134215<br>(p)LOC652301<br>(p)LOC100287990	**1,16,17,19**	1 [4]<br>14 [2]	NA	3A1[319]<br>3A2[31]<br>3B3[15]<br>3B1[13]<br>3B2[9]<br>3A5[7]<br>3A6[3]<br>3B5[2]<br>3A3[1]<br>3B4[1]<br>3A4[1]	3A1[26]<br>3A2[5]<br>3B3[1]
**1103279187477 NW_001840867.1**	100,705	23,927	7,299	73.7	(p)LOC101060863	**15**	15 [1]	20cen / 15cen<br>13cen 22cen	3A4[3]	3A4[7]
**1103279181301 NW_001839668.1**	53,723	20,196	5,117	85.2	none	**7**	7 [1]	7p11 / 2cen<br>1qh 16qh	3A2[14]<br>2B[5]<br>3A3[3]	3A2[7]<br>3A3[3]<br>2B[1]
**1103279180187 NW_001839391.1**	19,905	10,996	1,448	88.2	FAM138D (p)WASH3P	**20**	NA	NA	3A4[6]	3A4[3]
**1103276878137 (NA)**	12,911	10,320	3,657	86.2	NA	**13,21**	NA	NA	NONE	3A4[8]<br>3A5[1]
**1103276806665 (NA)**	21,097	10,102	3,713	89	NA	**2**	NA	NA	2B[34]	2B[37]<br>2A2[2]<br>3A2[1]<br>2A1[1]
**TOTAL**	3,882,801	1,287,186	617,748	86.2						
