# Supplementary material for: Genomic Characterization of Large Heterochromatic Gaps in the Human Genome Assembly
Source: PLoS Comput Biol. 2014 May 15;10(5):e1003628. doi: 10.1371/journal.pcbi.1003628 (PMC4022460; doi:10.1371/journal.pcbi.1003628)
Supplement: Table S10 — Experimental validation of HSat 2,3 associated unmapped scaffolds with reference to the Cytogenetic Resource of FISH-mapped, Sequence-tagged Clones. To provide further experimental validation of our unmapped scaffold chromosome assignments (listed in column 1), we compare our localizations with the reported mappings of 35 FISH-mapped clones available from the Cytogenetic Resource of FISH-mapped, Sequence-tagged Clones, or CytoBAC, (The BAC Resource Consortium (2001) Nature 409:953-958) and Levy et al. 2007). We highlight concordant predictions for 33/35 of the FISH assignments in red, indicating agreement in localization between our predictions (column 2) and those observed in previous experimental studies (column 3). (PDF) [file pcbi.1003628.s014.pdf]

Table S10. Experimental validation of HSat 2,3 associated unmapped scaffolds with reference to Cytogenetic Resource of FISH-mapped, Sequence-tagged Clones.

Scaffold ID	chrs assigned by WCS coverage (this study)	FISH assignment (CytoBAC, HuRef)
chr4_gl000193_random	13,14,21	9p12,4q35,14p11.1-14p11.2,9q12,4q28,21q11.1-21q11.2,13p11.1-13p11.2
chrUn_gl000212	13,17,19	2p11.2-2p12,22cen,13cen
AC237676.1	13,14,21,22	15p11.1-15p11.2,Yp11.1-Yp11.2,1q12,2p13,3q11.2,4p16.3,9p12,9q12,21p11.1-21p11.2,2q11.1-2q11.2,20q11.1-20q11.2,21p13,13p13,13p11.1-13p11.2,22p11.1-22p11.2,14p13,15p13,4p11-4q11,14p11.1-14p11.2
AC104301.2	20	18p11.3,15p11.1-15p11.2,4q35,3q13.3-3q21,16p11.1-16p11.2,Yp11.1-Yp11.2,9p12-9p13,21p11.1-21p11.2,20q11.1-20q11.2,13p11.1-13p11.2,22p11.1-22p11.2,14p11.1-14p11.2,3p12-3p13
AC146634.1	13,14,21	4q35,21q11.2,13p11.1-13p11.2,14p11.1-14q11.1
AC093749.3	14	3q28~29
FP885534.9	6	6q21,6q22
AC093614.4	7	7pter
AC010098.8	1	9p12,10p11.2,2p11.1-2p11.2,10q11.2,1q12,22q11.2,9q12-9q13,14q11.2,7p11.2,15q11.2,16p11.2,7q11.2,17p11.2,17q11.2
NW_001838589.2	1	1p12
NW_001838799.1	2	2p11
NW_001838877.2	3	3p14
NW_001838990.2	6	6q21
NW_001838996.1	6	6q25
NW_001838988.2	6	7p11 / 6q21
NW_001839024.1	7	7cen / 1cen, 2cen, 16cen, 15cen, 17cen
NW_001839181.1	9,19	9q12, 9p11 / 9q21, 4p16, 12q13, 13cen, 14cen, 15cen
NW_001838286.2	16	7q11, 16q11 / 1q12, 2p11, 22cen, 13/14/15cen
NW_001838666.1	20	5cen, 17cen, 19cen, 20cen / 10cen, 22cen
NW_001841138.1	20	15p13, 20cen, 22cen, 14cen / 2q13
NW_001840780.1	20	9cen, 13cen, 15cen, 20cen, 21cen, 22cen / 9q2, 14cen, 4q3
NW_001840853.1	20	20q11 / 9qh, 13cen, 14cen, 15cen, 21cen, 22cen
NW_001839651.1	3,14,16,17,19	2cen, 14cen, 22cen, 16cen / 9q21, 9cen, 15cen, 13cen, 10cen, 1q11
NW_001841150.1	1	1q11, 16q11.2, 7cen / 2p11.2, 3p21.3, 17p11
NW_001840871.1	14,22	1q12, 16p13, 15q11 / 14cen, 13cen
NW_001840306.1	13	13q34
NW_001841011.1	1	1q11, 16q21 / 2p12, 7q12
NW_001840146.1	14,22	1q42
NW_001841160.1	7	1q12, 7cen, 16q11 / 2cen, 9cen, 13cen, 14cen, 15cen, 22cen
NW_001840937.1	14,22	22p11.2 / 20cen, 9p13, 14cen, 15cen
NW_001839668.1	7	7p11 / 2cen, 1qh, 16qh
NW_001839364.1	3	6p12.1, 3p14
NW_001840016.1	7	7q11
NW_001840984.1	13	13q33
NW_001841149.1	9	9p11
