# Supplementary material for: Genomic Characterization of Large Heterochromatic Gaps in the Human Genome Assembly
Source: PLoS Comput Biol. 2014 May 15;10(5):e1003628. doi: 10.1371/journal.pcbi.1003628 (PMC4022460; doi:10.1371/journal.pcbi.1003628)
Supplement: Text S1 — Provides supplemental methods and corresponding references: simulating self-mate-pair frequencies for contiguous sequence domains, spectral clustering of feature vectors, and localization of unmapped scaffolds using WCS data. (PDF) [file pcbi.1003628.s015.pdf]

Supporting Online Material for

Genomic Characterization of Large Heterochromatic Gaps in the Human Genome Assembly

Nicolas Altemose[1,*], Karen H. Miga[1,#], Mauro Maggioni[2], and Huntington F. Willard[1]

[1] Genome Biology Group, Duke Institute for Genome Sciences & Policy, Duke University, Durham, North Carolina, United States of America.
[2] Department of Mathematics, Duke University, Durham, North Carolina, United States of America.

[*] Present Address: Department of Statistics, University of Oxford, Oxford, United Kingdom.

[#] Present Address: Center for Biomolecular Science & Engineering, University of California Santa Cruz, Santa Cruz, California, United States of America.

This PDF file includes:
Supplemental Methods
Supplemental References

SUPPLEMENTAL METHODS

Simulating self-mate-pair frequencies for contiguous sequence domains

To identify a suitable self-mate-pair frequency threshold for determining whether read clusters are physically distant in the genome, we simulated paired reads drawn from a contiguous domain of length L surrounded by a 200 kb buffer of "other" sequence on either side. The parameters for this simulation include:

1. ***R***, the mean high-quality read length in HuRef (~675 bp on reads with ≥75 continuous bases with phred>20)
2. ***C***, average high-quality base coverage in HuRef, where ~6.3x = (~29 million paired/unpaired reads) * (~675 mean high-quality bp/read) / (~3.1 billion bp in the haploid genome)
3. ***M***, proportion of high-quality reads with high-quality mate pairs (0.588)
4. ***D***, the categorical distribution of mate pair insert lengths used in the HuRef genome (Levy et al. 2007)[1].

The simulation draws N reads so that the average coverage by paired reads in the simulated region will equal the average coverage by paired reads in the genome ($N = M * (L+200000) * C / R$). Each read's start position is drawn uniformly along the length of the simulated region. An insert type is chosen according to the categorical distribution D, and insert size is determined according to a normal distribution with the insert type's mean size and standard deviation. If both the read and the mate pair occur within the domain of length L, they are each considered "self-paired reads." If a read but not its mate pair occurs within the domain, it is labeled as an "other-paired read." We calculate the final self-mate-pair (self-MP) frequency as the number of self-MP reads/(number of self-MP reads + number of other-paired reads). The simulation was performed for L with a range of 10,000 to 500,000 bp (in increments of 10,000 bp). For each value of L, we ran 1,000 iterations and reported the distribution of self-MP frequencies across these

iterations. As shown below, a self-mate-pair threshold of 0.8 eliminates >94% of simulated regions 100kb or shorter.

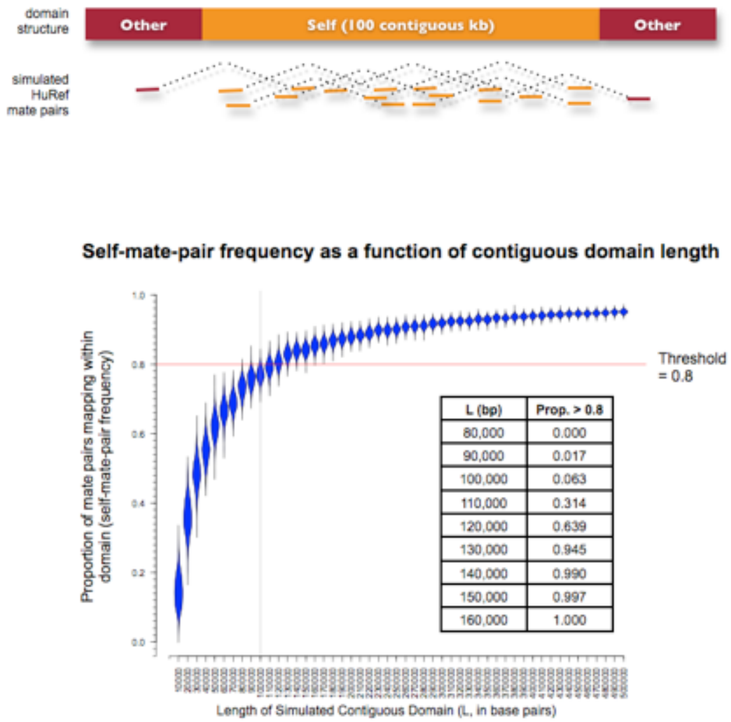

L (bp)	Prop. > 0.8
80,000	0.000
90,000	0.017
100,000	0.063
110,000	0.314
120,000	0.639
130,000	0.945
140,000	0.990
150,000	0.997
160,000	1.000

Results of self-mate-pair frequency simulations. Above is an illustration of the simulated region from which paired reads are drawn according the mate pair types in the HuRef genome. Below are violin plots representing the distribution of self-MP frequencies calculated for each domain size over 1,000 iterations of the simulation. The overlaid table shows the proportion of iterations of each domain size that pass a self-MP frequency threshold of 0.8.

Spectral clustering of feature vectors

In order to perform clustering of the high-dimensional HSat2,3 feature vector data, without *a priori* information on the shape and number of clusters, we use a hierarchical spectral clustering technique that also accounts for mate pair relationships, a natural extension of the standard spectral clustering technique (see e.g. [2-4]). The basic idea is to construct a graph whose vertices are the data points

(in this case, each point represents a feature vector for one HSat2,3 read) and whose edges connect neighboring data points, with an edge weight representing the similarity of the data points it connects (measured as the Euclidean distance between those vertices). The connectivity of such a graph can be used to define clusters in the data, i.e. regions that are highly intra-connected but with sparse inter-connections. The notion of conductance is one way to quantify this inter vs. intra connectivity; a subset of a graph with small conductance will have highly interconnected vertices but a relatively small number of edges exiting the subset. While finding subsets of low conductance is NP-hard, the problem may be relaxed into a much easier problem, that of computing a few eigenvectors of a Laplacian (or, alternatively, a random walk) operator on the graph. Such operators capture the properties of diffusions on the graph, which is intuitively and formally related to the ideas above: if diffusion of heat is started in a cluster with small conductance, it will take a long time before a significant portion of heat escapes the cluster, since there are so few edges connecting the cluster to the rest of the graph. A more formal description follows.

1.1. **Basic definitions.** We will need the following definitions (e.g., [5]) :

Definition 1.1. Let *(G,W)* be a weighted, undirected, connected graph in which G represents the vertices and W represents the edge weights*.* The **degree matrix** is the diagonal matrix defined by $D_{ii} = \sum_j Wij$. The **random walk** is the matrix $P := D^{-1}W$. The **normalized Laplacian** is the matrix $L := I - D^{-\frac{1}{2}}WD^{-\frac{1}{2}}$. .

In the definition above we are of course assuming that $\mathrm{D_{ii}} > 0$ for all i's, i.e. that there are no isolated vertices; if this is not the case we simply remove the isolated vertices. Also, we shall assume in what follows that the graph is connected, for otherwise the analysis below can be performed on each connected component.

Definition 1.2. Let us define the conductance of a subset S of a weighted graph (G, W) as

$$\phi_W(S) := \frac{\sum_{x\in S, y\notin S} W(x,y)}{\min\{\sum_{x\in S, y\in S} W(x,y), \sum_{x\notin S, y\notin S} W(x,y)\}} = \frac{\sum_{x\in S, y\notin S} W(x,y)}{\min(vol(S), vol(S^c))},$$

where $vol(S) = \sum_{x,y\in S} W(x,y)$

Note that $\phi_W(S)$ is small if the total weight of the edges in *(G, W)* that go across S and S^c is small compared to the total weight of the edges connecting points within S (or S^c, whichever is smaller).

While finding a set of vertices S with minimum conductance is NP-hard, there exist approximation algorithms; here we resort to a simple and commonly used algorithm, although there exist algorithms with better guarantees. Our first objective will be to partition the graph into two parts, each having small conductance. To do so, we let $L_{\varphi i} = \lambda_i \varphi_i$, for i = 0,1, ... and $0 = \lambda_0 < \lambda_1 \leq \cdots \leq \lambda_1 \leq \cdots$ be the eigendecomposition of L. Note that $\lambda_0 < \lambda_1$ since we assumed that G is connected. We think of φ_1, also called the Fiedler vector, as a function on G, since it is a vector of length equal to the number of vertices of G; it is well known that the two sets $S_+ := \{x \in G : \varphi_1(x) \geq 0\}$ and $S_- := \{x \in G : \varphi_1(x) < 0\}$ are connected, and have small conductance φ_+, φ_-. We split G into S_+ and S_-, and then we repeat the procedure on the corresponding induced subgraphs, in a hierarchical fashion, stopping when a split does not produce subsets of small enough conductance.

1.2. **Application to hierarchical clustering of 5-mer feature vectors.**

Definition 1.3. Let X represent the $D \times n$ matrix whose *(i, j)*-th entry is the frequency of the *i*-th 5-mer in the *j*-th read. We normalize X by standardizing the rows to have mean 0 and standard deviation 1.

A weighted graph (G, W_{sim}) is constructed from X as follows: the vertices of G are the columns of X, and the edge connecting vertices x*i* and x*j* is assigned weight $(\widetilde{W_{sim}})_{ij} := \exp(-\frac{\|x_i - x_j\|^2}{\epsilon_i \epsilon_j})$ if x_j is among the 50 closest points to x_i (otherwise no edge is created), and where ||x – y|| is Euclidean distance (in $\mathbb{R}^{2^5}$) and ϵ_i is the

distance between x_i and its 10-th nearest neighbor (see e.g. [4]). Since this construction leads to a non-symmetric weight matrix $\widetilde{W_{sim}}$ (as x_j may be among the 50 nearest neighbors of x_i without x_i being among the 50 nearest neighbors of x_j), we symmetrize $\widetilde{W_{sim}}$ and define the final weights to be $W_{sim} := \frac{1}{2}\,(\widetilde{W_{sim}} + \widetilde{W_{sim}}^T)$.

In addition to this similarity-driven graph, we have a second graph (G, W_{pen}) modeling exogenous information not captured by similarities. This graph may be much sparser than (G, W) if this information is given on a small subset of pairs of vertices. In our case the edges in (G, W_{pen}) represent paired read relationships, and are assigned maximal weight (1). This mate pair information is critical for selecting clusters that not only have different sequence patterns but also occupy physically distinct regions of the genome. We combine the two graphs (G, W_{sim}) and (G, W_{pen}) into *(G, W)* by combining the similarity matrices linearly: $W = W_{sim} + W_{pen}$. However, our measure of conductance when evaluating each iterative split will be entirely based on W_{pen}.

Our algorithm extends the standard recursive spectral bisection technique (e.g. [4-6]) and proceeds as follows. We construct the normalized Laplacian matrix L on *(G, W)* and compute the smallest 10 eigenvalues and eigenvectors of $L : L_{\varphi l} = \lambda_{l\varphi l}$ with $0 = \lambda_0 < \lambda_1 \le \lambda_2 \le \cdots$, where $\lambda_1 > 0$ as the graph is connected. For each eigenvector $\varphi_1, \ldots, \varphi_{10}$ and for a grid of values of δ in [min φ_l, maxφ_l], we consider the cluster $S_{l,\delta} := \{\, i : \varphi_l(x_i) > \delta \}$ and its complement $S^c_{l,\delta}$, and compute the corresponding conductance $\phi_{W_{pen}}(S_{l,\delta})$. We find the pair (l^*_1, δ^*_1) that minimizes $\phi_{W_{pen}}(S_{l,\delta})$. The resulting $S_{l*,\delta*}$ and $S^c_{l*,\delta*}$ are good clusters since they indicate that we found a cluster that is good not only with respect to similarity, since cuts based on the first eigenvectors of L tend to lead to clusters with small conductance ϕ_W, but also with respect to the exogenous conductance measure based on paired read information $\phi_{W_{pen}}$. We split the graph into $S_{l*,\delta*}$ and $S^c_{l*,\delta*}$ by severing the edges connecting these two subsets and repeat the splitting procedure on the corresponding induced subgraphs, i.e. computing the normalized Laplacians on each

of the two subgraphs, and using the corresponding eigenfunctions to split each cluster into two as above. We stop whenever the clusters have fewer vertices than a threshold v_{min}, or the conductance associated with a cut is smaller than a threshold φ_{min}.

A comparison of this spectral clustering approach with k-means clustering confirms the advantage of our method for identifying irregularly shaped clusters with high self-mate-pair frequencies, due to the fact that our method selects clusters with high nearest-neighbor connectivity and explicitly accounts for mate pair connectivity when making cluster divisions (see figure below).

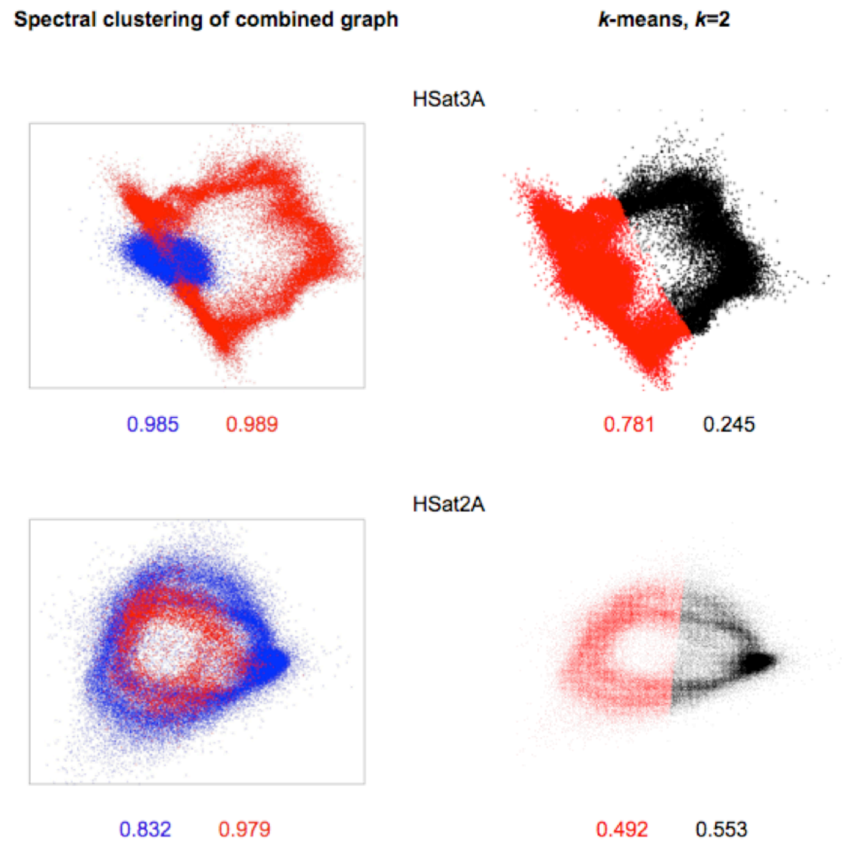

Comparison of unsupervised clustering approaches. Shown above are PCA projections illustrating the results of binary clustering of the HSat3A and HSat2A subgraphs, which both have ring-like topologies, using both our method (which clusters based on graph connectivity and explicitly accounts for mate pair information) and *k*-means. Below each plot are the self-mate-pair frequencies that result from these divisions. As shown, in cases of irregular topology which are common to our dataset, our clustering method outperforms *k*-means at identifying physically distinct clusters.

1.3. **Results on 5-mers.** We set the parameters as follows: υ_{min} = 1000, φ_{min} = 0.2. If we allowed splits with large conductance we would obtain more clusters. The value of φ_{min} was picked by calculating conductance values for simulated interspersed contiguous sequence arrays in a range of sizes (see above). This conductance threshold is expected to eliminate contiguous domains smaller than 100kb. In the

particular case of this data set, the 2nd eigenfunction is chosen most often for splitting, but occasionally higher order eigenfunctions (up to order 4) are chosen.

Localizing unmapped scaffolds using WCS data

Whole chromosome shotgun (WCS) read depth was determined over single-copy sites in the genome. In order to define unique regions on our set of unmapped scaffolds, we identified all non-RepeatMasked 24-mers for which no other 24-mer in the complete HuRef assembly (including unmapped scaffolds/contigs) is within a 2-bp edit distance. This requires the assumption that 24-mers that are present in a single copy in the HuRef assembly are present in a single copy in the genomes of all individuals (i.e. they are not sequence or copy number polymorphic, and the HuRef assembly contains all fixed copies of each 24-mer). Unique 24-mers were aligned to the full HuRef assembly (including all mapped and unmapped scaffolds) using BWA [6]. 24-mers with mapping qualities of 0 or with suboptimal alignments within an edit distance of 2 were discarded. We performed a similar analysis with GRCh37/hg19 chromosome reference sequences, utilizing available mappability tracks ("CRG Align 24", [7]).

Each single-copy 24-mer defines a small upstream region where an overlapping read alignment is likely to begin. Based on the empirical aligned read length distribution, we defined this 'valid unique region' for each 24-mer as the upstream 400 bp. Thus, for a given single-copy 24-mer with position *i*, a read alignment with a starting position in the interval (*i*-400, *i*) and with an exact match to that 24-mer has a roughly uniform probability of starting at any base in that interval. We can model the number of reads aligning to any valid position as a Poisson distribution, and thus we can also model the total number of reads aligning to valid regions on any given scaffold with a Poisson distribution. With these conditions, we evaluated

18,779 unmapped HuRef scaffolds (containing ≥100 valid base pairs and ≥ 1 mapped WCS read).

We expected unmapped scaffolds to localize to more than one chromosome if they contain recently duplicated sequences that may have been collapsed in the assembly [8,9]. To predict single or multiple chromosome localizations, we used a binary "localization vector" of length 24, with each position indicating whether it localizes to a particular chromosome (1) or not (0). There are 2^{24} - 1 = 16,777,215 possible valid states that this vector can take. However, any given scaffold is unlikely to map to most or all chromosomes. To reduce our search space, we only performed likelihood calculations for localization vectors whose sum is between 1 and 5 (55,454 total), effectively setting the prior to 0 for all localization vectors with more than 5 chromosome assignments. For each of the 18,779 unmapped scaffolds, we define a vector of length 32 with each entry indicating the number of reads from one of the 32 WCS samples with unique alignments starting on valid positions on that scaffold.

Let $\boldsymbol{Y}$ be an 18,779 x 32 matrix containing these vectors as rows.
Let $\boldsymbol{n}$ be a vector of length 18,779 indicating the total number of valid positions on each scaffold.
Let $\boldsymbol{X}$ be a 24x32 matrix containing the number of reads from each WCS sample with unique alignments starting on valid positions on each of the 24 chromosomes.
Let $\boldsymbol{m}$ be a vector of length 24 indicating the total number of valid positions on each chromosome.
Let $\boldsymbol{\Theta}$ be a 55,454 x 24 matrix containing all possible localization vectors.
Let $\boldsymbol{C} = \boldsymbol{\Theta}(\boldsymbol{X}/\boldsymbol{m})$, a 55,454 x 32 matrix representing the probability for a particular localisation state that a valid position is an alignment start for each WCS sample.
Let $\boldsymbol{P}$ be a 55,454 x 1 column vector containing the prior probability of each localization state. We used a prior that sets uniform total probability to each

subset of localization vectors summing to 1, 2, 3, 4, or 5 (with each subset's total probability distributed uniformly to all of its localization states).

Let $Y_{sw} \mid \Theta_{tw} \sim Poisson\ (n_s C_{lw})$, where l is the localization state (55,454 total), s is the scaffold index (18,776 total) an dw is the WCS sample index (32 total). Thus the likelihood can be expressed as:

$$L(\Theta_l|Y_s) \propto \prod_{w=1}^{32}[(n_s, C_{lw})^{Y_{sw}} e^{-n_s C_{lw}}]$$

with the log likelihood as:

$$l(\Theta_l|Y_s) \propto \sum_{w=1}^{32}[Y_{sw}\log(n_s C_{lw})) - n_s C_{lw}]$$
$$= \sum_{w=1}^{32} Y_{sw}\log(n_s) + \sum_{w=1}^{32} Y_{sw}\log(C_{lw}) - n_s \sum_{w=1}^{32} C_{lw}$$

For each unmapped scaffold we calculated analytically the likelihood of each of the 55,545 possible localization states given the available WCS alignment data. We then multiplied each likelihood by each localization state's prior, and we rescaled their posterior probabilities to sum to 1. For each scaffold we then calculated the marginal posterior probability for each chromosome by summing the posterior probabilities of all localization states containing that chromosome. A marginal posterior probability above a threshold of 0.9 resulted in an assignment to that chromosome.

We repeated this analysis for a different set of unmapped scaffolds, belonging to the set of "decoy sequences" compiled by the 1000 Genomes Project as a set of nonredundant read mapping targets not present in the GRCh37 assembly [10]. Together with GRCh37, these decoy sequences form a reference called hs37d5. This reference includes GRCh37 unmapped scaffolds plus HuRef scaffolds that are not present in hg19 (including large mapped and unmapped scaffolds in the HuRef assembly), BAC/fosmid sequences present in Genbank, and ALLPATHS-LG contigs from the NA12878 assembly. We used hs37d5 for identifying unique 24-mers and mapping WCS reads, and we report localization results for all scaffolds containing at

least ≥100 valid base pairs and ≥ 1 at least one mapped WCS read (see Dataset S3). For HuRef scaffolds localized by both analyses, we report the localization probabilities from the analysis done with the HuRef assembly, as it is more comprehensive than the hs37d5 assembly owing to its inclusion of smaller contigs

Evaluating WCS-based HSat2,3 subfamily mapping results

We compared our WCS-based mapping results (Table S7) to published experimental mapping results of available HSat2,3 clone sequences (Table S1) and HSat2,3 sequences in hg19 (Table S8), as well as published mapping locations of oligonucleotide probes specific to HSat2 (found primarily on chromosomes 1, 2, 10 and 16 but also on 7, 15, 17, and 22) or HSat3 (found primarily on chromosome 9 but also on chromosomes 1, 5, 10, 13, 14, 15, 17, 20, 21, and 22) (Tagarro et al. 1994). Importantly, we found that the oligonucleotide used by Tagarro *et al.* to identify HSat3 is only found in the HSat3B clade (not in the HSat3A clade, which includes the dominant families on the Y chromosome). Our WCS model predicts HSat3B5 localization to chromosome 8, which contradicts published mapping results. This discrepancy likely owes to the fact that the only available WCS sample for chromosome 8 is mixed with chromosome 9, and chromosome 9 is also represented in another WCS sample from a different donor. Because our WCS model does not account for array size variability between WCS donor individuals, our model will likely assign any 'extra' HSat3B5 from one individual onto another chromosome present in the mixed sample (i.e. chr8). Our model also predicts minor HSat3A6 localization to chromosome 7 (~94 kb), which would contradict hybridization-based methods finding HSat3A6 to be specific to males. This could represent a real array on chr7 that is polymorphic or simply too small to be reliably detected by hybridization-based methods (as is likely the case for many small domains of HSat2,3 sequence found on hg19).

REFERENCES

1. Levy S, Sutton G, Ng PC, Feuk L, Halpern AL, et al. (2007) The diploid genome sequence of an individual human. PLoS Biol 5: e254.
2. Ravi Kannan SV, and Adrian Vetta (2004) On clusterings: good, bad and spectral. J ACM 51: 497-515.
3. A. Ng MJ, and Y. Weiss. (2002) On spectral clustering: Analysis and an algorithm. Advances in neural information processing systems 2: 849-856.
4. Zelnik-Manor L. PP (2004) Self-tuning spectral clustering. Advances in neural information processing systems: 1601-1608.
5. Chung FRK (1997) Spectral Graph Theory. CBMS Regional Conference Series in Mathematics Volume 92.
6. Li H, Durbin R (2010) Fast and accurate long-read alignment with Burrows-Wheeler transform. Bioinformatics 26: 589-595.
7. Derrien T EJ, Marco Sola S, Knowles DG, Raineri E, Guigó R, Ribeca P. (2012) Fast computation and applications of genome mappability. PLoS ONE 7: e30377.
8. Bailey JA, Gu Z, Clark RA, Reinert K, Samonte RV, et al. (2002) Recent segmental duplications in the human genome. Science 297: 1003-1007.
9. She X, Jiang Z, Clark RA, Liu G, Cheng Z, et al. (2004) Shotgun sequence assembly and recent segmental duplications within the human genome. Nature 431: 927-930.
10. Genovese G, Handsaker, R. E., Li, H., Kenny, E. E., & McCarroll, S. A. (2013) Mapping the Human Reference Genome's Missing Sequence by Three-Way Admixture in Latino Genomes. The American Journal of Human Genetics 93: 411-421.
11. Choo KHA, Earle E, Vissel B, Filby RG (1990) Identification of two distinct subfamilies of alpha satellite DNA that are highly specific for human chromosome 15. Genomics 7: 143-151.
12. Nakahori Y, Mitani K, Yamada M, Nakagome Y (1986) A human Y-chromosome specific repeated DNA family (DYZ1) consists of a tandem array of pentanucleotides. Nucleic Acids Res 14: 7569-7580.
13. Moyzis RK, Albright KL, Bartholdi MF, Cram LS, Deaven LL, et al. (1987) Human chromosome-specific repetitive DNA sequences: novel markers for genetic analysis. Chromosoma 95: 375-386.
14. Cooke HJ, Hindley J (1979) Cloning of human satellite III DNA: different components are on different chromosomes. Nucleic Acids Res 6: 3177-3197.
15. Jeanpierre M (1994) Human satellites 2 and 3. Ann Genet 37: 163-171.
16. Jackson MS, Mole SE, Ponder BAJ (1992) Characterisation of a boundary between satellite III and aiphoid sequences on human chromosome 10. Nucleic acids research 20: 4781-4787.
17. Jackson MS, Slijepcevic P, Ponder BA (1993) The organisation of repetitive sequences in the pericentromeric region of human chromosome 10. Nucleic Acids Res 21: 5865-5874.
18. Jeanpierre M, Weil D, Gallano P, Creau-Goldberg N, Junien C (1985) The organization of two related subfamilies of a human tandemly repeated DNA is chromosome specific. Human genetics 70: 302–310.

19. Bandyopadhyay R, McQuillan C, Page SL, Choo KH, Shaffer LG (2001) Identification and characterization of satellite III subfamilies to the acrocentric chromosomes. Chromosome Res 9: 223-233.
20. Choo KH, Earle E, McQuillan C (1990) A homologous subfamily of satellite III DNA on human chromosomes 14 and 22. Nucleic Acids Res 18: 5641-5648.
21. Higgins MJ, Wang HS, Shtromas I, Haliotis T, Roder JC, et al. (1985) Organization of a repetitive human 1.8 kb KpnI sequence localized in the heterochromatin of chromosome 15. Chromosoma. pp. 77-86.
22. Choo KH, Earle E, Vissel B, Kalitsis P (1992) A chromosome 14-specific human satellite III DNA subfamily that shows variable presence on different chromosomes 14. Am J Hum Genet. pp. 706-716.
23. Vissel B, Nagy A, Choo KH (1992) A satellite III sequence shared by human chromosomes 13, 14, and 21 that is contiguous with alpha satellite DNA. Cytogenet Cell Genet 61: 81-86.
24. Deininger PL, Jolly DJ, Rubin CM, Friedmann T, Schmid CW (1981) Base sequence studies of 300 nucleotide renatured repeated human DNA clones. J Mol Biol 151: 17-33.
25. Tagarro I, Fernandez-Peralta AM, Gonzalez-Aguilera JJ (1994) Chromosomal localization of human satellites 2 and 3 by a FISH method using oligonucleotides as probes. Hum Genet 93: 383-388.
26. 1000 Genomes Project Consortium (2010) A map of human genome variation from population-scale sequencing. Nature 467: 1061-1073.
